# Yellow Mealworm and Black Soldier Fly Larvae for Feed and Food Production in Europe, with Emphasis on Iceland

**DOI:** 10.3390/foods10112744

**Published:** 2021-11-09

**Authors:** Runa Thrastardottir, Hildur Thora Olafsdottir, Ragnheidur Inga Thorarinsdottir

**Affiliations:** 1 Faculty of Agricultural Sciences, The Agricultural University of Iceland, IS-311 Hvanneyri, Iceland; runa.th@simnet.is; 2 Faculty of Civil and Environmental Engineering, The University of Iceland, IS-107 Reykjavik, Iceland; htho4@hi.is

**Keywords:** mealworm, black soldier fly larvae, insect farming, novel protein, Europe, food, feed, Iceland

## Abstract

Insects are part of the diet of over 2 billion people worldwide; however, insects have not been popular in Europe, neither as food nor as a feed ingredient. This has been changing in recent years, due to increased knowledge regarding the nutritional benefits, the need for novel protein production and the low environmental impact of insects compared to conventional protein production. The purpose of this study is to give an overview of the most popular insects farmed in Europe, yellow mealworm, *Tenebrio molitor*, and black soldier fly (BSF), *Hermetia illucens*, together with the main obstacles and risks. A comprehensive literature study was carried out and 27 insect farming companies found listed in Europe were contacted directly. The results show that the insect farming industry is increasing in Europe, and the success of the frontrunners is based on large investments in technology, automation and economy of scale. The interest of venture capital firms is noticeable, covering 90% of the investment costs in some cases. It is concluded that insect farming in Europe is likely to expand rapidly in the coming years, offering new proteins and other valuable products, not only as a feed ingredient, but also for human consumption. European regulations have additionally been rapidly changing, with more freedom towards insects as food and feed. There is an increased knowledge regarding safety concerns of edible insects, and the results indicate that edible insects pose a smaller risk for zoonotic diseases than livestock. However, knowledge regarding risk posed by edible insects is still lacking, but food and feed safety is essential to put products on the European market.

## 1. Introduction

The act of eating insects is called entomophagy and comes from the Greek terms “entomos”, meaning “insects”, and “phagein”, meaning “to eat” [1]. Humans have eaten insects as a part of their diet for millennia all around the world [2] and today insects are part of the diet of over 2 billion people worldwide. About 2000 species of insects are eaten in the world today. Most of them are eaten in Central and South America (679 species), and 549 species of insects are consumed in Mexico alone. Entomophagy is also widely practiced in Africa (524 species), Asia (349 species) and Australia (152 species). However only 41 species of insects are eaten in Europe [3]. In 2019 it was estimated that 9 million Europeans consumed insects [4], which is about 1.2% of the European population in 2019 [5]. In comparison, a survey performed in Kinshasa in the republic of Congo in 2003 reported that 70% of the city’s inhabitants consumed insects [6]. Additionally, about 25% of the world population consumes insects today [3,7]. However, the consumption of insects is declining in Asia but has also been reinvented in new forms and contexts, according to Andrew Müller [8]; this might be caused by several reasons as a form of modernity and globalization.

In spite of the high consumption of insects in the world, Europeans have abandoned entomophagy a long time ago and consider it to be a primitive behaviour [9]; however, this is slowly changing [4]. There are some speculations on why Europeans have abandoned this practice but one of the most likely reasons is the difference in the weather. Europe is in the temperate zone where insect species are smaller than in the tropics and insects are unavailable in the wintertime [10]. However, the ancient Greeks and Romans ate insects [11] and cockchafer (type of a beetle) soup was consumed in Central Europe even until the 20th Century [12]. There is an increased interest for edible insects in Europe today [10], both as a source of food and feed, which can be traced back to a 1975 publication by Meyer-Rochow [13], who urged Food and Agriculture Organization of the United Nations (FAO) and World Health Organisation WHO to take up the idea and support the use of edible insects as a food item for humans and animals. This interest increased rapidly when insects became regarded as a novel food in the European Union (EU) in 2015. The number of research papers on mealworms in Europe were 65 between 2012 and 2015 and 133 between 2016 and 2019 [3].

The global population is projected to increase to approximately 10 billion by 2050. Although the food produced could feed 10 billion people [14], 10% of the world still suffers from hunger every day [15]. Only two third of all food produced is consumed and the rest represents a huge waste of natural resources [14]. To counter this problem and to stop world hunger before 2030, new ways of producing and using food is required [15,16] along with alternative food and feed sources [17]. One way to reduce waste and world hunger is to grow insects on organic waste for the production of animal feed or food [6]. Studies have shown that both mealworms [18] and black soldier fly (BSF) larvae can be grown on waste [19], along with several other insect species [20].

Another thing to consider is the environmental effects of food production; the current food system is responsible for 80% of deforestation, 29% of all greenhouse gas emission, and agriculture uses 34% of all land on the planet, and withdraws 70% of freshwater and is responsible for 68% of animal extinction [14]. Growing insects requires less greenhouse gas production, water use and use of land per kg food produced than livestock, and thus can be produced in an environmentally sustainable manner [18].

Insects as feed are also considered to have less of an environmental impact compared with most-used protein sources today, soybean and fishmeal [21]. For example, oceans are overfished and 20% of all wild caught fish is used for aquaculture feed, fishmeal [14]. Most feed protein sources such as soybean and fishmeal are imported into the EU [17], with South America being the biggest producer of fishmeal [6] and South America and the United States being the biggest producers of soybean [22]. However, some fishmeal is produced in Iceland, Ireland, Denmark, Faroe Islands, Norway, United Kingdom, Estonia and Spain [23] and some soybean is produced in Ukraine, Russia, Italy, Serbia, France, Romania, Hungary and Austria [22]. The recent high demand has led to the high prices of these feed today [6], and the prices are expected to increase even more [17].

In 1975, edible insects were suggested to be able to counteract food shortage by increasing the use of them as food and feed [13]. Edible insects have been used as an alternative protein source for both humans and animals and research has shown that insects have a good nutrient value for humans, poultry, pork [18] and for aquaculture [24].

In this article, the production of mealworm and BSF larvae as food and feed in Europe is studied. Companies that have already started farming these species were contacted and asked key questions about their operations, investments and current status. The article also discusses the obstacles of using insects as feed or food in Europe and the food security related to insect farming. European regulations and how they are developing are presented as is how insect farming supports the Sustainable Development Goals (SDG) and European strategies. Moreover, the consumer acceptance of insects for feed and/or food is estimated. Finally, the article looks deeper into the situation in Iceland.

## 2. The Benefits of Breeding Insects

Edible insects have been shown to have nutritional, ecological and economic advantages, and increased insect farming is considered to promote increased food security across the world. Insect products are also considered to be beneficial for the health and welfare of livestock and they could lead to reduced antibiotic use in livestock production [25].

### 2.1. Health Benefits

#### 2.1.1. Nutritional Value as Food

Edible insects are considered to be a valuable source of nutrients, with a high amount of energy, protein and fats. They are high in amino acids and monosatured fatty acids, which meets the requirements of humans. Besides being high in nutrients, edible insects are also rich in certain vitamins and minerals [25] and have a high content of fiber compared to livestock, as seen in Table 1 [26,27]. It has also been reported that insect protein has as many nutritional benefits as milk proteins [28] and that edible insects might decrease cholesterol levels in humans by 60% [29].

Insect species are highly variate in crude protein content, but on average, the crude protein content of edible insects ranges between 35–60% dry matter, which is higher than plant protein sources, including cereal, soybeans and lentils and the insects with a higher amount provide more protein than even meat and chicken eggs [30]. Live mealworm include about 20% protein, while dried mealworm includes about 53% protein [31]; the average protein content of BSF larvae is between 38–48% [27]. According to Liu et al. [32], the crude protein content of BSF varies between diverse lifecycle stages. In 1 day larvae, 14 days larvae, prepupae, pupae and adults it is 56.2%, 39.2%, 40.3%, 45% and 43.9% respectively. However, crude protein content is overestimated when using a nitrogen to protein conversion factor of 6.25, mainly due to the presence of chitin [33], which is not digestable, but insect protein digestibility is estimated to be between 77–98% [31].

Insect fat content varies between species, sex, reproduction stage, season, diet and habitat [31]. The average fat content of edible insects is between 2–50% dry matter [34]. The average fat content of mealworms is between 19.12–34.54% [18] and the average fat content of BSF larvae is between 15–35% [27]. Mealworms are reported to have a high level of polyunsaturated fatty acids [17]; however, studies have shown that edible insects are in general low in omega-3 fatty acids and have a high omega-6/omega-3 ratio. This can be changed by adding omega-3 fatty acids to insect diets [35]. Insects are reported to have a higher content of energy, sodium and saturated fat than conventional livestock. A high content of sodium and saturated fat in food can lead to over-nutrition=linked diseases such as heart diseases. However, insects tend to have a very high micronutrient content, especially in the micronutrients that are known to be deficient in many areas where food insecurity is high. This shows that meat products may be nutritionally preferable to certain insects in the context of overnutrition, and that severeal insects are potentially superior to meat in the context of undernutrition. However, nutritional composition of a product does not say everything about its effect on human health [36].

It has been reported that nutritional quality of edible insects varies greatly depending on the insect diet [18,27,31]. BSF larvae have been reported to be able to accumulate both lipid- and water-soluble nutrients from their diet, and BSF reared on brewery waste or a mixture of fruit and vegetables have been shown to have a higher protein content than BSF reared on fruit or winery by-products [27]. Futhermore, mealworms reared on plant waste have a higher protein content and a lower fat content than mealworms reared on a cereal-based diet [18] and, as mentioned earlier, adding omega-3 fatty acids into insect diets can decrease the omega-6/omega-3 ratio [35]. Additionally, it has been reported that processing methods can affect the nutritional quality of edible insects [27,37]. In a study by Nyangena et al. [27], it was reported that heat processing increases the protein content and decreases the fat content of BSF larvae.

**Table 1:** Nutritional level in raw products, mealworms and BSF larvae compared to ground pork, ground beef, ground chicken, and farmed Atlantic salmon.

	Mealworm	BSF Larvae	Pork	Beef Cattle	Poultry	Salmon	Daily Value ^1^
**General nutritional profile**	[18,26,36,38]	[27,39,40,41,42]	[36,43]				[36,44]
Crude protein (g/100 g)	15.80–18.60	12.0–36.3	15.41–31.69	15.76–29.46	17.44–23.28	19.84–25.44	50
Fat (g/100 g)	10–26.6	12.25–29.8	4–33	3–30	8.1–13.9	6.34–13.42	65
Crude fiber (g/100 g)	0.68–1.29	7.9–8.1	0	0	0	0	
Energy (kcal/100 g)	152–268		121–393	121–332	143–198	142–208	2000
Crude ash (g/100 g)	1.13	3.9–15.8	0.79–1.49	0.7–1.71	1.17–1.57	1.13–3.26	
**Fatty acids**	[36,38,45]	[41,46]	[36,43]				[36,44]
Saturated fat (g/100 g)	2.58–8.97	6.14–23.50	1.42–11.31	1.48–11.75	0.8–4	0.98–3.05	20
Monounsaturated fatty acids (g/100 g)	3.79–14.29	1.49–8.60	1.89–15.33	1.13–14.17	3.61–4.88	2.10–4.18	
Polyunsaturated fatty acids (g/100 g)	1.10–3.17	2.07–6.39	0.66–4.32	0.22–0.70	1.51–2.08	2.54–4.55	
**Vitamins**	[26,36]	[41]	[36,43]				[36,44]
Vitamin A (µg/kg)	57–205		0–50	0–70	0	120–690	15,000
Niacin (mg/kg)	40.7–46.5		35.97–110.5	33.82–74.85	48.7–76.5	80.45–100.77	200
Pyridoxine (mg/kg	6.9		1.67–7.17	2.78–4.35	5.12–5.38	6.36–9.44	
Riboflavin (mg/kg)	8.1–8.7		1.8–4.88	1.51–2.5	1.25–3.02	1.35–4.87	17
Folat (mg/kg)	1.55		0–0.06	0.040–0.21	0.01–0.02	0.25–0.34	
Biotin (mg/kg)	0.43						
Thiamin (mg/kg)	1.1–2.4		2.71–9.28	0.3–0.8	0.68–1.21	2.07–3.40	15
Vitamin B_12_ (µg/kg)	1.3		6.4–23	19.7–29	5.1–5.6	28–32.3	
Vitamin C (mg/kg)	99.0–120		0–23	0	0–20	0–39	600
Vitamin D (IU/kg)	<80		40–340	20–80		4410–5260	
Vitamin E (mg/kg)	33	53.3–248.8	2.6–4.7	0–4.3	2.7–3.9	11.4–35.5	
**Minerals**	[18,26,36,38,47]	[41,42,48]	[36,43]				[36,44]
Iron (mg/kg)	9.61–245	100–630	7–15.1	15.4–32.9	7–10	3.4–10.3	180
Zinc (mg/kg)	33.8–117.4	42–300	19.1–35.9	35.7–71.5	14.7–19.2	3.6–8.2	
Magnesium (mg/kg)	620–2027	2100–5610	160–270	140–290	210–280	270–370	
Calcium (mg/kg)	156–435	5360–61,620	60–200	50–410	60–120	90–150	10,000
Phosphorus (mg/kg)	2640–7061	6800–13,220	1610–2610	1320–2670	1780–2340	2000–2560	
Sodium (mg/kg)	225–3644	890–2500	555–940	525–960	600–895	440–610	24,000
Potassium (mg/kg)	3350–9480	10,200–18,790	2440–4280	2180–4700	5220–6770	3630–6280	
Copper (mg/kg)	8.3–20	7.5–34.25	0.33–1.31	0.5–1.08	0.62–0.65	0.45–3.21	
Manganese (mg/kg)	3.2	190–730	0.10–0.13	0.09–0.22	0.16	0.11–0.21	
Selenium (mg/kg)	0.12	0.1–1.2	0.25–0.49	0.13–0.23	0.10–0.14	0.24–0.47	

^1^ Daily values from the US Food Labelling Guide [44]. All are daily reference values (DVRs) except for vitamins and minerals, which are recommended daily intake (RDI) value.

#### 2.1.2. Nutritional Value as Feed

Insects are a natural part of the diet of some animals including pig, poultry and fish [49] and edible insects and can be used as a protein source in feed for these species along with other protein sources such as soybean meal and fishmeal [18,21,40,49,50]. Mealworms contain essential amino acid compositions sufficient to meet the dietary requirements of trout, where mealworms can replace fishmeal in the diet. However, mealworms lack sufficient methionine to meet the essential amino acid requirement of salmon, poultry and humans [21]. Studies have shown that edible insects can improve the growth rate and digestibility of poultry and pigs compared to other protein sources. Increasing the mealworm content to 15% in poultry diets increased the body weight and the daily feed intake of chicken in one study. In the same study, increasing mealworm content to 6% in pig diets increased the body weight, average daily gain, average daily feed intake and gain to feed ratio of weaning pigs [18]. Additionally, a study by Rawski et al. [40] showed that the replacement of fishmeal with BSF larvae can have positive effects on Siberian sturgeon growth performance.

#### 2.1.3. Chitin

Besides protein and fat, insects contain fiber, mainly in the form of chitin. Chitin is a natural polysaccharide, which is probably one of the most abundant biopolymers in nature and is the second most abundant biomass in the world after cellulose. It plays a structural role in many organisms, including fungi, crustaceans, mollusks, coelomates, protozoa and green algae [51]. The composition and amount of chitin in insects can vary between species and developmental stages. Most of the chitin can be found in the exuviate (shed). Although chitin can play a role in the pathogenesis of asthma and allergies, it is considered to have potential positive effects on the immune system.

Chitosan, one of chitin’s derivatives, is produced by deacetylation and can be a valuable by-product for biomedical use. Primex Iceland, an Icelandic company, has been producing chitosan from shrimp shells (*Pandalus borealis*) from the North Atlantic Ocean. They have been producing healing spray and gel for external use both for humans and animals, but also as a dietary supplement for weight management [52]. The composition and the amount of chitin varies with the species and developmental stages. The chitin contents of mealworms are considered to be between 4.92–13.0 g/100 g, with an average of 6.41 g/100 g [18]. The proportion of chitin in BSF larvae is similar, or is around 5.69–7.95 g/100 g [53].

#### 2.1.4. Prebiotics and Probiotics

Prebiotics are defined as a fiber that stimulates the growth of preexisting good bacteria in the gut, but probiotics as a live microbial is a feed supplement that beneficially affects the host. Research has been carried out on whether chitin plays a role as a prebiotic of animal origin. It seems that chitin’s derivatives, such as chitosan, chitin-glucan (GC), and chitin oligosaccharide (NACOS), show better results in the modulation of gut microbiota (GM) by enhancing the growth of beneficial bacteria and by inhibiting the growth of pathogenic bacteria along with anti-inflammatory effects. However, bacteria can have different roles in different species. The ratio between the types of bacteria seems to play an important role in human health. The derivatives showed more prebiotic activity when carried by low protein-containing food. Research has shown that fewer antibiotics are needed when insects are used as feed [51,54].

Probiotic bacteria in mealworm diets could be beneficial, because larvae are processed whole, so the residual microbiota is carried to the end consumer. It is estimated that the microbiota is up to 10% of the total insect biomass. The bacteria can also produce B vitamins and can decrease the need for antibiotics by stimulating the host immune system [17].

### 2.2. Environmental Benefits

#### 2.2.1. Greenhouse Gas Emission

Edible insects have less of a negative effect on the environment than other livestock forms. Insect farming produces less greenhouse gas than livestock [25,55] and uses less land and water [25], as seen in Table 2. Moreover, insects are often considered to be environmentally friendly because their farming may have a low feed conversion ratio (FCR); however, this varies between insect species and the feed. For example, the FCR for mealworm ranges between 2.2 to 5.3, while the FCR for nymphal stage of *Acheta domesticus* ranges between 1.08 to 4.5 [56]. The FCR for edible insects can vary depending on the feed used [57]. When reared on an optimal diet, mealworms convert feed as efficient as poultry and the nitrogen use efficiency is higher than traditional livestock [17].

In a study by Oonincx et al. [55], greenhouse gasses (CO_2_, CH_4_ and N_2_O) and ammonium (NH_3_) emissions by five species of insects (including mealworms) were measured and compared to greenhouse gas and NH_3_ emissions of cattle and pigs. The results showed that four of five insect species produced much less greenhouse gas than pigs and only 1% of greenhouse gas compared to ruminants. All of the insect species produced less NH_3_ than cattle and pigs. Furthermore, mealworms do not produce methane (CH_4_), contrary to pigs and cows. However, the true environmental effect of mass rearing of insects has not been determined [57] and a complete lifecycle analysis for edible insect species is lacking [55].

Breeding insects for feed is considered to be more environmentally friendly than the protein sources used for food and feed today, soybean and fishmeal [21]. A study by Smetana et al. [58] showed that the production of 1 kg of BSF larvae resulted in less land use, less CO_2_ production and less water use than the production of both soybean meal and fishmeal. Fishmeal is produced through the overexploitation of fish in the oceans [14,25], as 20% of all wild caught fish are used for fishmeal [14]. Additionally, studies have shown that feeding chickens [59] and aquaculture [60] with mealworms can reduce these species’ FCR.

**Table 2:** Environmental effect of mealworms and BSF compared to livestock.

Species	Total Greenhouse Gas Emission (g/kg Body Mass) ^1^ [55]	FCR Dry Matter ^2^	Land Use m^2^/kg [61]	Water Footprint m^3^/Edible Ton [62]
Mealworm	0.45	1.6–2.1 [63]	3.56 [64]	4341
BSF	N/A	1.8 [65]	2 [66]	N/A
Pork	2.09–28.22	4.04–6.4 [67,68]	17.36	5988
Beef cattle	6.23–>7.53 ^3^	18.9–25 [67,68]	326.21	15,415
Poultry	3.0–5.1 [69]	2.67–3.3 [67,68]	12.22	4325

^1^ CO_2_, CH_4_, N_2_O, NH_3_; ^2^ FCR: Feed Conversion Ratio; ^3^ No data for N_2_O.

#### 2.2.2. Waste Management and Plastics

Almost 100 years ago, the idea of processing organic waste by using fly larvae was proposed. Studies have shown that several fly species are suited for the biodegradation of organic waste, e.g., BSF and house flies (*Musca domestica*) [70]. Furthermore, insects like mealworms can be used to biodegrade organic waste and plastic to proteins [18]. BSF larvae seems to be able to degrade a large variety of organic waste, ranging from food waste, agri-industry co-products, animal waste to meat-based products [71], aquaculture sludge [48], substrate containing up to 50% seaweed [41], and BSF larvae, and are also commonly found in rotten fruits and plant residues [70]. However, the composition of the substrate is important as it has a major effect on BSF development, survival, nutritional composition and the substrate bioconversion rate [71]. The composition of the substrate also affects food and feed safety, as it might contain metals and pathogens that can accumulate in the larvae [48].

A study by Tsochatzis et al. [72] showed that mealworms can be used to degrade plastics when reared on plastics, barley and water. The results indicated that plastic compounds do not bioaccumulate in mealworms and that a very low content is released into the frass. However, the consumption of plastics caused the mealworms more metabolic stress in comparison to their typical diet.

#### 2.2.3. Environmental Risks

There are some concerns regarding the effect of a possible escape of edible insects into the environment and becoming locally invasive species to natural and production systems in non-native countries [57]. In Iceland, an environmental risk assessment for BSF was performed before receiving license from the Icelandic authorities for import and trials. The results showed that BSF poses no threat to the local insect environment, and it is highly unlikely that a wild population can form if an escape will happen. This is due to BSF being a tropical species that is not likely to survive in the cold climate of Iceland [73]. However, there is evidence that BSF could be established in Europe, especially with climate change making the establishment of many more non-native species more likely [57]. The northernmost region where wild BSF has been recorded is in the Czech Republic [74].

## 3. Food Security

For novel food to be placed on the European market, it must be safe, meaning it must not have any harmful effects on health or be unfit for human consumption according to the EU general food law [75]. This is also important regarding the novel products used for animal feed, as this is the most important factor to guarantee the sustainable production of safe and affordable animal proteins [76]. To prevent possible harmful effects, the risk regarding novel food and feed products must be known, and there must be techniques in place to prevent those risks [75]. The risk associated with edible insects can be allergies, toxins and pathogens [73].

### 3.1. Allergies

Food allergy is an adverse immunological response to a foreign substance. Further research is needed, but some studies report the potential allergy risk posed by mealworms or other insects. One study showed that mealworm proteins cross-reacted in vitro with IgE produced by patients who were allergic to house dust mites or crustaceans (crabs, lobsters, crayfish etc.) in response to tropomyosin (a structural protein found, e.g., in the cytoskeleton). Heat processing of the product reduces the allergic response, but it still exists. A double-blinded placebo study in humans showed that mealworm allergy is most likely in people allergic to shrimp, with a potentially severe outcome. A safety assessment of freeze-dried mealworm powder in rats showed no adverse effects, allergy or toxicity [17].

Another potential risk is that insects may carry mold that can cause allergic reactions. This can affect the workers in production as well as the consumers. Additionally, allergens from the feed (e.g., gluten) may end up in the insect that is consumed [77].

### 3.2. Toxicity

Use of insects as food and feed have raised questions about toxicity. Mealworms may contain defence substances, such as toxins produced by the exocrine and defensive glands. Focus has been placed on benzoquinones, which are secreted into the abdominal cavity in adult beetles and have toxic effects, but these findings refer to *T. molitor* beetles and not to larvae. Other hazards are contained in aflatoxin, mycotoxin, heavy metals, organic pollutants, plasticisers, flame retardants and others. It seems that different species show a different accumulation behaviour, e.g., BSF accumulates cadmium, but mealworms accumulate arsenic in the larval body and therefore it is important to keep a regular monitoring of contaminants in their feed. It is important also to keep track of every step of the production [78,79]. According to the EFSA Panel, the toxicity studies of mealworm from the literature did not raise any safety concerns, but noted that the larvae should be reared separately from the adult beetles [33,80]. Eleven applications for other species are pending for safety evaluation by EFSA [29].

### 3.3. Antinutrients

Anti-nutrients or antinutritional factors (ANFs) are, contrary to nutrients, compounds that reduce the absorption of nutrients. They are found in common foods such as whole grains, soybeans, spinach, broccoli, tea and coffee, even in chocolate. Glucosinolates, one anti-nutrient found in mustard and cabbage, can prevent the absorption of iodine and thus disturb thyroid function and cause goiter. They are therefore also known as goitrogens, and this is of special concern if there are pre-existing hypothyroidism. Lectins (hemagglutinins), found in the vast majority of organisms, can reduce the absorption of calcium, copper, iron, phosphorus and zinc. They are carbohydrate-binding proteins and can cause the agglutination of red blood cells. Raw legumes and whole grains contain higher levels of lectins, so it is important to take into account how the food is processed. Phytates (phytic acid) in whole grains, seeds, legumes and even nuts can decrease the absorption of iron, zinc, magnesium and calcium. Oxalates, found, for instance, in tea and chocolate, can prevent the uptake of calcium by forming calcium oxalate and tannin in tea, and coffee and legumes can decrease iron uptake. The effect of anti-nutrients differs between people’s health and metabolism and which food they otherwise consume and when. Interestingly, anti-nutrients are also thought to have benefits for health, such as phytates, which have been found to lower cholesterol and to increase balance in blood sugar as well as having antioxidant effects. There are limitations however, because it is difficult to study the role of anti-nutrients in various diets and their levels differ in how the food is processed [81]. Edible insects are mostly herbivorous, as they feed on plants and plant parts. Plants synthesize different types of secondary metabolites for their self-preservation, and these secondary metabolites are known as allelochemicals and accumulate in the bodies of plant matter-ingesting insects. Insects contain a wide variety of antinutrients, which is likely caused by the different chemical compositions of plants on which the insects feed [82]. According to Turck et al. [33], the levels in whole dried mealworm larvae are comparable to the occurrence levels in other food substances. The development of rearing techniques of edible insects under controlled conditions can minimize, or even avoid the contamination of insects with antinutrients. Furthermore, it has been reported that processing methods can help to remove antinutrients and other unhealthy components [82].

### 3.4. Zoonosis

Microbiological hazards associated with insects as food and feed are either part of the insect’s lifestyle and gut flora, or are introduced via human contact, through farming and processing. The insect’s gut flora is essential for the metabolism and survival of the insects. The gut flora varies depending on the species and it includes bacteria, viruses, and fungi. Most of mealworms’ and BSF larvae’s gut flora is not pathogenic to humans and other animals; however, microbiota introduced during farming and processing possess a greater risk to humans and other animals [73].

#### 3.4.1. Bacteria

There have been few studies into the microbial content of mealworms and BSF larvae, and its effect on food safety. These studies indicate that there is a high level of bacteria on the surface and in the gut of these insects [83,84,85,86,87,88,89,90].

Bacteria pathogenic to insects are considered harmless to humans and other vertebrates, since insects are so phylogenetically different. Therefore, bacterial hazards for humans and vertebrates will mainly originate from the insect microbiota, related to rearing conditions, handling, processing, and preservation [73]. Mealworm’s microbiota consists mostly of Proteobacteria, Firmicutes and Actinobacteria with Propionibacterium being the most abundant taxa [83]. BSF larvae microbiota consists mainly of Proteobacteria and Firmicutes with *Providencia*, *Klebsiella* and *Bacillus* being the most abundant taxa, while the microbiota of prepupae consists mainly of Bacteroidetes, Proteobacteria, Firmicutes and Actinobacteria, with many taxa dominating the microbiota, e.g., *Providencia*, *Myroides*, *Proteus* and *Morganella*, that can all act as opportunistic pathogens and may carry drug resistance [84].

Currently, no microbiological criteria exist specifically for insects sold as food; however, hygiene criteria for the processing of minced meat described in EU Regulation EC No. 1441/2007 can be used for insects. According to these criteria, the limit for the total aerobic count is 5.7 log cfu/g [83,85], the average total aerobic count in fresh and powdered mealworms and BSF larvae was higher than this limit (>8 log cfu/g on average) in most studies [83,85,86,87]. The current food hygiene criteria include *Salmonella enterica (S. enterica)*, *Listeria monocytogenes* (*L monocytogenes*), *Escherichia coli* (*E. coli*), *Staphylococcus aureus* (*S. aureus*) and *Bacillus cereus* (*B. cereus*) [88]. According to several studies, mealworms and BSF larvae do not act as a vector for *S. enterica* [73,86,87,88,89]; however, in a study done by Raimondi et al., 2020, [84] on BSF, *S. enterica* was detected in some samples of prepupae. It is believed that BSF larvae possess antimicrobial capacities that make them able to reduce pathogenic bacteria such as *S. enterica* and *E. coli* [87,90]. In most studies, *L. monocytogenes* and *E. coli* are not detected in the mealworms BSF larvae [86,87,88,89]; however, these bacteria can be detected in these species if they are reared on substrates contaminated with *L. monocytogenes* [91,92] and *E. coli*, but *E. coli* will be reduced in the larvae [92]. *Staphylococcus aureus* was detected in mealworms in a study done by Stastnik et al., 2021, [89] and coagulase-positive staphylococcus was detected in BSF prepupae in a study done by Raimondi et al., 2020 [84]. However, both studies had coagulase-positive staphylococcus under the contamination limit. All staphylococci detected in two other studies were coagulase-negative, meaning that no *S. aureus* was detected [87,88].

*Bacillus cereus* seems to be one of the biggest hazards regarding the use of edible insects as food [87,88]. Some strains of *B. cereus* produce toxins that cause emesis or diarrhoea [88]. To produce toxins, the density of *B. cereus* is believed to have to be around 4–5 log cfu/g. Some studies suggest that an even lower density is needed, but the density of *B. cereus* in BSF larvae in one study went up to 3.8 log cfu/g [87]; however, in another study where *B. cereus* was investigated on BSF pupae, one dried sample and one powdered sample exceeded the limit—one over 5 log cfu/g and another over 6 log cfu/g [88]. In a small study on mealworms in the Netherlands, 93% of all samples had less than 2 log cfu/g of *B. cereus* [73]. In another study, the median values of *B. cereus* in mealworm samples were in general under 4 log cfu/g, with two outliers between 4–5 log cfu/g [93]. Since neither do all *B. cereus* produce toxins nor are all non-cereus bacilli toxin free, toxic gene profiling may be a better diagnostic tool to estimate the true hazard [88].

*B. cereus* is also a spore-forming bacteria [87]. A high content of bacterial endospores has been found on mealworm and BSF larvae [73,86,87]. Endospores and toxins produced by *B. cereus* are heat- and processing-resistant [85,87,94]. Endospores can also germinate and produce toxins when food is not cooked, cooled and stored properly [94]. Bacterial endospores highly differentiate in number between different rearing batches from the same company [83,86,87,89]; in one study, the number of endospores in mealworms varied between 1.7 log cfu/g to 5.0 log cfu/g [86]; in another study focused on mealworms, the endospores detected were between <1 log cfu/g to 3.5 log cfu/g [84]. In a study on BSF larvae reared on different substrates, the endospores varied between 3.7 log cfu/g to 7.5 log cfu/g [87]. It is unclear why different samples reared within similar conditions have such a high variance in endospore content [83], but it is believed that the rearing substrate and insect species influence this [73,87]. *Bacillus cereus* is widely spread in soil water and in plants [87], and insects farmed on soil are believed to be more likely to include bacterial endospores [83]. According to these studies, BSF larvae seem to be more contaminated with *B. cereus* and endospores than mealworms. This could be caused by the different substrate used for these two species. While mealworms are normally fed with various flour types, and are often complemented with carrots, BSF larvae are often fed with soil and food waste that usually contains high levels of *B. cereus* and endospores. According to Wynants et al., 2019 [87], one strategy to avoid food poisoning through BSF larvae is to only use substrates that do not carry *B. cereus*. However, this would reduce the economic positivity and sustainability of BSF rearing. Therefore, more research into *B. cereus* and endospores-contaminated insects and how to reduce the risk is needed.

Other pathogenic bacteria that have been detected in mealworms and BSF larvae are *Clostridium* spp. [83,84,87,89] and *Campylobacter* sp. [84,90,95] However, only low levels of *Clostridium perfringens* have been detected in these studies, with an average concentration under <1 log cfu/g [84,89]. High levels of *Campylobacter* sp. have been isolated from BSF prepupae [84,90] and the lesser mealworm (closely related to mealworm) has been shown to be able to infect poultry through ingestion; however, *Campylobacter* sp. is only active in the larva for 3 days after exposure [95].

Even though zoonotic pathogens found in the substrates used to grow insects could lead to insects acting as a vector for these bacteria, no active replication seems to occur in insects. However, zoonotic pathogens are widely known to be able to replicate in farmed animals, e.g., *Salmonella* [73].

#### 3.4.2. Virus

Most viruses on insects are insect-specific and are not pathogenic for vertebrates [73]. Insects’ viral pathogens are considered to be safe for humans and are approved in some cases as biocontrol agents in agriculture. The biggest problem these viruses cause is a financial burden to the insect farms, since viruses associated with insects are only pathogenic to the insects themselves [96] and may cause a loss in production. Vertebrate viruses taxonomically related to insect viruses are unable to replicate in insects, and are not actively transmitted by insects as vectors to vertebrates [73]; therefore, these viruses are not considered to lead to a health risk in humans and other vertebrates [96].

Today, there are no studies on the pathogenicity of insect-specific viruses in humans, but it is believed that the specificity of insect viruses is mainly limited to the species taxon and are unable to replicate in vertebrates. Due to the lack of comparable viruses between insects and vertebrates, the risk of recombination and reassortment of an insect-specific virus strain leading to a new mammalian specific virus strain, as was the case of Swine flu and COVID-19, is almost non-existent. Therefore, an increased consumption of insects is likely to reduce the risk of a new pandemic in the future [97].

However, viruses in insects that are called arthropod-borne viruses, or arboviruses, can cause human diseases and can replicate in both insect vectors and vertebrates. Known diseases caused by arboviruses are, e.g., West Nile disease, dengue, rift valley fever, haemorrhagic fever, and chikungunya [96]. There is no evidence that such viruses occur in insects used for food and feed [73]. Arboviruses are believed to originate from insect-specific viruses, which indicates that an evolutionary process might lead to novel insect origin pathogens in the future following the introduction of insects into the diet [97].

Another issue is that insects can also act as passive vectors of vertebrate viral diseases, where the virus does not replicate in insect vectors, but is rather carried by the vector to the host [73]. Adenovirus, norovirus, rotavirus, hepatitis E, and hepatitis A could possibly be introduced with a substrate in insect farms and could be transferred further through the production [73,96]. However, there is a lack of information relating to the likelihood of such transmission from feedstock through residual insect gut contents. Studies have shown that adenovirus, norovirus, and hepatitis A could survive in untreated manure and litter for at least 60 days at 20 °C and 4 °C, and other temperatures were not tested [73].

It has been concluded that the risk of edible insects acting as a passive vector of COVID-19 is extremely low, which demonstrates that edible insects should not be a reservoir for viral diseases with epizootic potential [97]. In the case of insects acting as passive vectors of vertebrate viruses, processing and cooking will reduce the risk of transmission in most cases [73,96].

#### 3.4.3. Fungi and Yeast

Fungi, such as yeast and mold, are a part of edible insects’ normal microbiota. These microorganisms produce spores and can easily spread to different environments and can contaminate food. Fungi causes the deterioration of food, nutritional losses, discolouration, and an off flavour and are the major organisms responsible for food spoilage. Some species of fungi are pathogenic to vertebrates and can produce toxins, e.g., mycotoxin [94]. Studies have shown that insect-specific pathogenic fungi pose a small risk to humans and other vertebrates. However, these fungi have occasionally caused diseases in immunosuppressive individuals [73,97]. Insects might also be carriers of fungi and yeast pathogenics to vertebrates and a considerable amount of fungi and yeast have been found in fresh, freeze-dried and frozen mealworms [73]. It has been reported that dried mealworms can be carriers of *Penicillium* spp., and *Mucor* spp., while fat from BSF larvae can carry *Aspergillus* spp., and *Cryptococcus neoformans*. *Aspergillus* spp., *Penicillium* spp., and *Cryptococcus neoformans* have been found in many insects and can cause opportunistic infections in humans; however, no direct infection after consuming insects has been recorded [89].

Good hygiene in the entire production chain will reduce the risk of fungi infection introduced during farming processing and storage [73]. However, if hygiene is not acceptable, studies have shown that a short-blanching of 10–40 s can considerably reduce fungi [85,94]. Incorrect storage conditions of feed intended for insects can lead to fungi formation in the feed and this type of fungi may form mycotoxins [89]. According to studies done on the accumulation of the mycotoxin in mealworms and BSF larvae, very low-levels of mycotoxins accumulate in these species. Mealworms and BSF larvae fed with feed spiked with high mycotoxin levels showed an accumulation well below the limit value [89,98,99,100] in food and feed, according to Commission Regulation (EC) No 1881/2006, Commission Recommendation 2006/576/EC, and Directive 2002/32/EC. The mycotoxins studied where aflatoxin B1 [89,99,100], deoxynivalenol [89,98,100], ochratoxin A [89,100] and zearalenone [100]. These results indicate that mycotoxins should not be a concern regarding the use of mealworms and BSF larvae as food or feed.

#### 3.4.4. Parasites

Insects have been known to be able to infect humans with parasites through consumption for a long time. In 1871, it was discovered that a common parasitic disease in Russia was caused by the consumption of a raw beetle larvae that was an intermediate host for this parasitic disease [101]. Most studies on parasites in insects are related to non-European areas and insects harvested in the wild but the results from these studies suggest it to be a problem. However, the risk will be reduced in farmed insects with a strict control over the environment [73]. In a study done on edible insects as a vector for parasites, several parasitic species were detected in mealworms that can be pathogenic to humans and animals. *Cryptosporidium* was the most prevalent pathogenic parasite detected in mealworms and it was found in 16% of all analysed mealworm farms and in 12% of all samples. *Cryptosporidium* was found in the gastrointestinal tract and other parts of the mealworm’s body. It is possible that mealworms can infect humans with *Cryptosporidium* aerogenically, and infection can occur on farms that are lacking in proper hygiene regarding contact with insects. Other pathogenic parasites detected in mealworms were *Isospora* spp., *Balantidium* spp. *Entamoeba* spp. *Cestoda*, *Pharyngodon* spp. larva, *Physaloptera* spp. larva, *Spiroidea* spp., and *Acanthocephala* spp. However, some of these parasites came with mealworms, which were delivered from outside of Europe and some of the farms were guilty of unethical practices that would not be accepted if the insects were farmed for food or livestock feed [102]. Another parasite that has been detected in mealworm larvae is *Toxoplasma gondii* [103].

The results from a study done on endoparasites within invertebrates used as a live feed for wild caged birds indicates a low risk for parasite transmission associated with mealworm consumption by birds [104].

Not as much research has been done on BSF larvae working as vectors for parasites like mealworm. However, there is evidence that BSF larvae can act as a vector for *Eimeria* and *Ascaris suum* [105].

According to EFSA Scientific Committee [73], insects reared in a properly managed closed farm environment would lack all the hosts necessary for the completion of parasite life cycles. Beside proper management before consumption, freezing and cooking, would further eliminate potential parasitical risk.

Canthariasis is the invasion of a living beetle larva on a living or dead organism, making them act as parasites themselves. Different species of beetle larvae lead to different pathological changes and clinical signs; the main categorization of canthariasis relies on the invasion location in the host. Mealworms rarely cause canthariasis, but there are some reported cases in the world. Mealworm larvae usually lead to gastric canthariasis [106], which can affect both humans [107] and animals through the ingestion of eggs or larvae. The clinical signs of gastric canthariasis can be nausea, vomiting, stomach-ache, abdominal bloating, loss of appetite, weight loss, and diarrhoea, resembling intestinal parasite infection. In extreme cases, the larvae penetrate the intestinal organs and invade other organs. Gastric canthariasis can lead to death if untreated [106]. Other organs mealworm larvae are known to invade are umbilicus and tonsils and there is a one known case of mealworm larva invading bladder and causing urinary canthariasis in humans [107]. Mealworms feeding live to animals and humans therefore contain a danger, but if the larvae are killed before consumption, the danger will be neglectable as long as eggs are filtered away from the larvae used for consumption.

#### 3.4.5. Prion

Prion disease or transmissible spongiform encephalopathies are naturally occurring infectious protein-misfolding disorders that characterise the accumulation of misfolded protein aggregates in the brain. Prion diseases affects several mammals, and they are always fatal, an example of this diseases is Creutzfeldt-Jakob disease (vCJD) in humans, scrapie in sheep, bovine spongiform encephalopathy (BSE) in cattle and chronic wasting disease (CWD) in deer and elk. On rare occasions, prion diseases can be transmitted between species [108]; therefore, there exist concerns relating to the possibility of prion diseases being transmitted from insects through food or feed.

There is no evidence that there exists a special prion disease in insects, since no gene encoding prion or prion-related proteins have been reported in insects [109,110]. Therefore, mammalian prion cannot replicate in insects and insects are not considered to be possible biological vectors of mammalian prion diseases [73]. However, research has shown that insects can possibly act as a mechanical vector of prion disease. Mites from Icelandic sheep farms with a known scrapie infection were able to infect mice via intracerebral injection [111,112] and larvae of *Sarcophaga carnaria* (*S. carnaria*) fed with brain material from scrapie-infected hamsters were able to infect hamsters through an oral route at different stages and after death [109]. Additionally, studies have shown that *Drosophila melanogaster* (*D. melanogaster)* can act as a mechanical vector for prion diseases [113]. Since replication of prions are not considered possible in insects, the number of prions in the substrate used to feed the insect affects the total prion infectivity of insects and cannot be higher than in the substrate. The substrate strongly influences the possible risk of prion disease transmission and must therefore be controlled at insect farms to counter this problem. The substrate used to feed the insects should not have a ruminant nor a human origin, but according to EFSA regarding the risks related to prion-derived diseases, the risk in non-processed insects is expected to be equal or lower than the proteins of another animal origin, as long as the insects are fed on substrates that do not harbour material of a ruminant or human origin [73]. However, no research has been done into the transmission of prion disease through the consumption of mealworms or BSF larvae.

**Figure 1:**
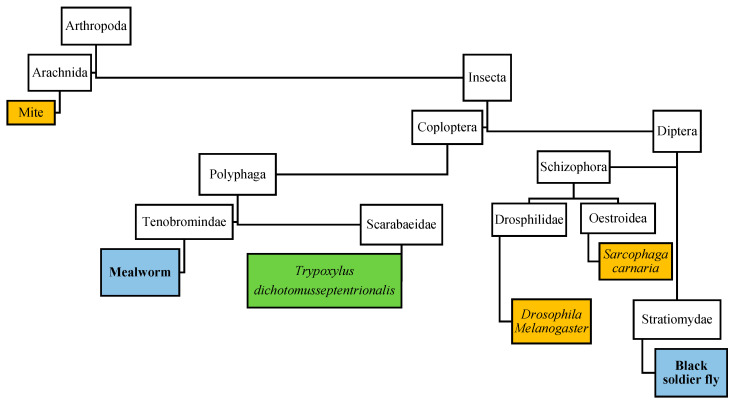
Relationship between mealworms and BSF (colored blue) to species that have been shown to be able to act as vector for prions (colored yellow) and to species that might possibly have a cure for prion diseases.

One study shows that insect haemolymphs might have an anti prion effect, haemolymph from the beetle, *Trypoxylus dichotomus septentrionalis* (*T. d. septentrionalis*) showed anti-prion activity on a special strain of prions after being heated at 70 °C for 3 h [114]. Mealworms are genetically more related to *T. d. septentrionalis* than to *S. carnaria*, *D. melanogaster* or mites. Figure 1 shows the relationship between the insect species.

### 3.5. Food and Feed Safety Management

For food to be placed on the European market, it must be safe, meaning that it must not have harmful effects on health or be unfit for human consumption according to the EU general food law [75]. For this, there must be good hygiene practice in place (GHP) through the whole production chain and an ability to trace products. Production sites must be easy to clean and be constructed to eliminate pests and cross-contamination and must not contain hazardous chemicals [115]. Insects reared in a properly managed closed farm are less likely to act as a vector for parasites, since they would lack all host necessity to complete the lifecycle [73]. There is also a need for sufficient ventilation that can reduce air contamination and controlled temperature and humidity appropriate for the insect species. All equipment, vehicles, boxes, and tools used in the production site must be dedicated solely to insect-rearing activities and be cleaned thoroughly between batches [115].

Employees must be aware of hygiene requirements and be trained in GHP and other hygiene systems provided by the company. There has to be a separate area for staff to change to work clothes and staff are also required to use appropriate protective tools, e.g., people that have direct contact with products must wear gloves and people that work in the breeding chambers must use masks [115].

Insect producers in the EU must only use substrates that are accepted as feed for farmed animals within the EU. The substrate has to be traceable and of appropriate hygiene standards and must not contain any chemical contaminants. Insect producers should carry out regular checks of incoming substrate materials and substrates must be stored in dry, temperature appropriate and hygienic conditions [115]. Substrate control is an important part of safety management regarding insect breeding, because the substrates ingested can have a strong influence on insects’ microbiota [90,91,92,116].

It is recommended to register rearing conditions and to test insects regularly for pathogens and chemicals [115]. 24 h before harvesting mealworms, it is recommended to remove them from the substrate for intestine cleaning [85,115,117,118]. This is performed because of the high microbial content in the insects gut [73,85]; however, there has not been documented benefits from this procedure [75]. When harvesting, foreign materials must be removed along with dead insects and frass. Chilling insects under controlled temperatures before harvesting has been reported to be beneficial for both mealworms and BSF larvae; it results in the maintenance of product properties and avoids microbial contamination [115].

Several killing methods have been researched regarding the food security of insects [85,119,120]. Farmed mealworms are often killed with blanching, boiling vapor, or freezing, while farmed BSF larvae are often killed with mincing and blanching. Blanching is performed by plunging insects into hot water, which will instantly kill the insects and destroy the microbial flora [115], and then they are often chilled by putting into clean water [115,119]. For mealworms, blanching is found to be the most successful heating method as it considerably reduces the bacterial content and fungi [85,94,117,119]; however, blanching is not sufficient to kill bacterial endospores [85,87,94,118]. As there is high endospore content in soil [87], which can be used as a substrate, and substrate influences the insects microbiota [90,91,92,116], management of the substrate is important, but the use of classical feed additives or fermentation has been shown to reduce spore forming [87]. Drying and acidifying techniques of insects are also promising to reduce endospores [118]. After blanching, mealworms can be stored in a refrigerator for 6 days without substantial microbial growth [119]. Not all time and temperature combinations will result in a sufficient reduction in microbial pathogens, and it is recommended to monitor the temperature used. An inadequate heat treatment can lead to bacterial proliferation. Another killing method, freezing, must be performed below 5 °C; however, most freezers operate at −20 °C and the appropriate freezing time to kill varies from species to species. Freezing has been shown to maintain the insects’ nutritional value until they are further processed [115].

After killing, it has been shown that drying insects is important to reduce potential microbial, chemical, and allergenic hazards [121]. Freeze drying [115] and heat-based dehydration methods are used [115,119] and effective processing methods have been shown to further reduce the microbial load [73]. Sometimes, insects are processed through grinding for powder formation or fractioning, e.g., extracting chitin. These processing methods must be performed under GHP, and the grinding machine must be cleaned regularly. Water activity and storage temperature must also be appropriately monitored to reduce potential microbial contamination under processing and packaging [115].

Insects and insect-derived products must be stored in a close, clean and appropriate place and regard the product specification. There must be a prevention of accumulation of organic material and sampling plan for analysis of hazards for incoming raw materials and the outgoing product. If there is a transportation of food and feed products derived from insects, the same hygiene standards must be applied through the transportation as in other parts of the production chain [115].

One of the most important parts of the production chain is the packaging of insects, as it contributes to the condition that products will be in when they reach the consumers. Therefore, good hygiene, environment, security, and quality practices must be performed to ensure the safety of a product. The packaging must be clean and must not contain any chemical, physical or microbiological hazards. After the product is in the package, it must be closed immediately, and the operator must ensure that no external source of contamination is included. To prevent allergenic hazards, the product must be labelled with potential allergens in the product and a list of ingredients [115].

To simplify the control of potential hazards that can come up, there has been a developed system for the food industry that is called Hazard Analysis Critical Control Point (HACCP) [75]. However, no specific HACCP plan exists for the rearing of insects, but breeders have been working according to HACCP with company-specific approaches [73]. A properly designed HACCP can have control over all parts of food production that might pose a risk [122] and can prevent, eliminate and reduce to acceptable levels, microbial, chemical and physical hazards [115]. An HACCP system must be considered in the design, organization, and management of food production sites, along with the design of premises and equipment and a product-traceable system. With insects being considered as food or feed, it is necessary to guarantee their safety, but one of the main limitations of developing the insect farming industry involves guaranteeing the safety of the products. Therefore, manufactures must implement an HACCP plan to limit the risk for consumers’ health [75].

When developing an HACCP, several things must be in place. There must be conducted hazard analysis for the production; Critical Control Points (CCP) must be determined; and there must be established critical limits and a system to monitor the CCP. Additionally, there must be established corrective actions when monitoring indicates that a particular CCP is not under control. There must also be established procedures of verification to confirm that the HACCP system is working effectively [115] and that should be documented [75].

Hazard analyses consist of hazard identification and an evaluation of the likelihood and severity of those hazards. It also consists of finding preventive measurements for these hazards. Hazards associated with insects as food and feed can be of a pathogen, chemical, allergenic and physical origin [115]. Whole insects, processed insect powder, and insects for food or feed can include different hazards [75].

Critical Control Points are defined as steps where control can be performed to prevent, eliminate or reduce a food and/or feed hazard to an acceptable level [75]. All CCPs require control measures, monitoring procedures, responsible staff, records and identified measurable critical limits to determine safe and unsafe conditions. In the insect industry, CCPs can be chilling, blanching, metal detectors in process lines [115], cooking after killing and hot drying [75] where the critical limits could be related to, e.g., temperature, pressure, time, water activity and pH [115]. When level outside of the critical limits are measured at one CCP, examples of corrective actions are, e.g., destroy the batch, readjust the temperature or time or restart the step [75].

## 4. Insect Farming in Europe

Many insect farming companies have emerged in the last few years. Some countries have had strict rules and regulations, but in other countries, a considerable experience is in marketing insects, i.e., for human consumption. This year, a step was taken by the European Commission by allowing yellow mealworm (*T. molitor)*. Some countries, e.g., Belgium, Czech Republic, Denmark, Finland, the Netherlands and the UK, allow companies to keep selling whole insect-based products as long as applications for the species were made before 1st of January 2019 (transition period) [123].

### 4.1. Insect Farming in Europe

Insect farming is a growing industry in Europe [4] and it is a new business for Europeans [124]. Today, it is allowed in Europe to use insect-derived proteins, whole insects and insect-derived fats in pet food, feed for fur animals, and in aquaculture. Additionally, whole insects and insect-derived fats are allowed in feed for pigs and poultry [125,126,127]. Currently, in 2021, insect protein for feed is mostly produced as a pet food and for aquaculture [124]; however, this is believed to be about to change in the next few years in light of the recent authorisation of insect proteins for poultry and pigs on the 17 August 2021 [128]. By the end of the decade, new regulatory developments are expected to play a key role in increasing the production of insects and insect-derived products. Several tonnes of processed insect protein were produced in 2020 and the production of insects for feed is estimated to increase rapidly in the coming years. It is forecasted that the production of insect proteins for feed will reach 1 million tonnes of insect meal by 2030 [124]. At the same time, there has been a rapid change in the dietary habits of Europeans [4] and the willingness of consumers to try insect-based food is increasing [4,129,130,131]. This can be linked to an increased knowledge regarding the nutritional benefits and environmental effects of insects, alongside an increased willingness to consume environmentally friendly food [4,131,132,133,134,135]. This change in attitudes around food and growing demand for high protein food for sport nutrition, dietetic food or food supplements creates new opportunities for the production of insects as food. Currently, the use of insect-derived ingredients in food is low, but it is estimated to increase rapidly in the next few years [4] following mealworms being newly authorized for human consumption [33] and new insect products are expected to be authorized by the end of 2021 and by early 2022 [136,137,138]. In 2019, 500 tons of insect-based products for human consumption was produced in Europe, but the market for edible insect-based food products is estimated to produce 260,000 tonnes by 2030. Additionally, in 2019, 9 million Europeans consumed insects and insect-derived products, but by 2030 it is estimated that insects and insect-derived products will reach 390 million European consumers [4]. For insects to be a suitable alternative animal feed and for human consumption, insect farmers need to be able to produce large quantities of insects and insect-derived products and to have a steady production with sufficient quality. To be able to reach this level, insect farmers need to invest in capacity to offer satisfying quantity within costs that can compete with conventional animal feed used today, along with meat [139]. Increased availability of insect-derived products will lead to a decrease in prices [124]. The International Platform of Insects for Food and Feed (IPIFF) is an EU non-profit organisation that represents the interests of the insect production sector towards EU policy makers, European stakeholders and citizens. Within IPIFF that are 79 members [140], with 45 of them being insect companies in Europe today [141].

### 4.2. The Law in Europe

**Figure 2:**
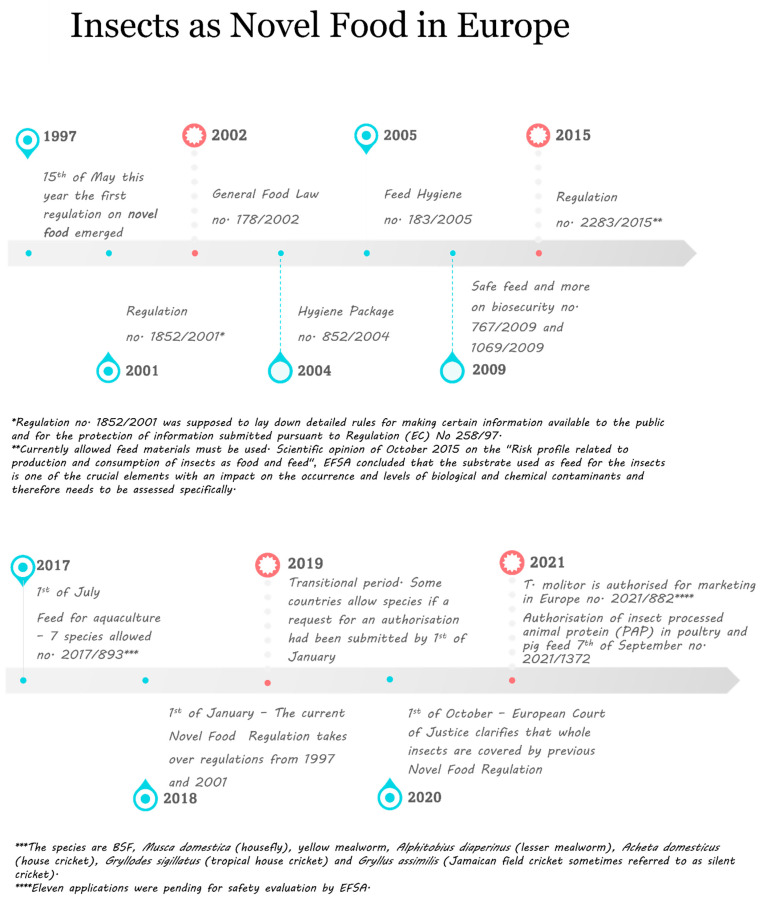
Insects as a Novel Food in Europe, timeline showing the main regulatory changes since 1997. Source: The European Commission [34,150,151,152].

European law on insects in food and feed must strike the right balance between innovation and safety. The International Platform of Insects for Food and Feed (IPIFF) is an EU non-profit organization originally created in 2012 with its main mission to promote the wider use of insects and by advocating for EU legislative frameworks. The term ‘Novel Food’ is defined as food that has not been consumed to a significant degree by humans in the European Union before 15th of May 1997, when the first Regulation on novel food appeared. The main components are protein, fat and fiber (chitin). Since then, many new regulations have emerged (see Figure 2). Regulation no. 2283 from 2015 took over the regulations from 1997 and 2001 to update and develop guidance for applications for authorization of novel foods to the Commission, who may request a risk assessment from the European Food Safety Authority (EFSA) [142]. The marketing of dried mealworm, recently or on the 3rd of May 2021, got authorization to be placed on the market as a novel food. The European Food Safety Authority (EFSA) though, concluded that the consumption of the yellow mealworm may potentially lead to an allergic reaction, especially in individuals with pre-existing allergies to dust mites and crustaceans. Therefore, it is important to identify it on the food label. Toxicological and nutritional factors were also evaluated. The toxicity studies from the literature did not raise safety concerns and the consumption is not nutritionally disadvantageous [33].

Another EU Regulation, no. 2017/893, from 1st of July 2017, allowed a list of seven insect species to be included in the formulation of feeds for aquaculture. The species are BSF, *Musca domestica* (housefly), mealworm, *Alphitobius diaperinus* (lesser mealworm)*, Acheta domesticus* (house cricket)*, Gryllodes sigillatus* (tropical house cricket) and *Gryllus assimilis* (Jamaican field cricket sometimes referred to as a silent cricket) and even silkworm (*Bomby mori*) [142]. Previously, the addition of insects in feed for animals was not allowed due to potential prion-derived diseases. All other insect-based products were considered “Novel Food”, and fell under EU regulation no. 2015/2283, where specific application to the European Commission is needed followed by EFSA scientific evaluation, before putting the product on the market as previously mentioned [143].

**Figure 3:**
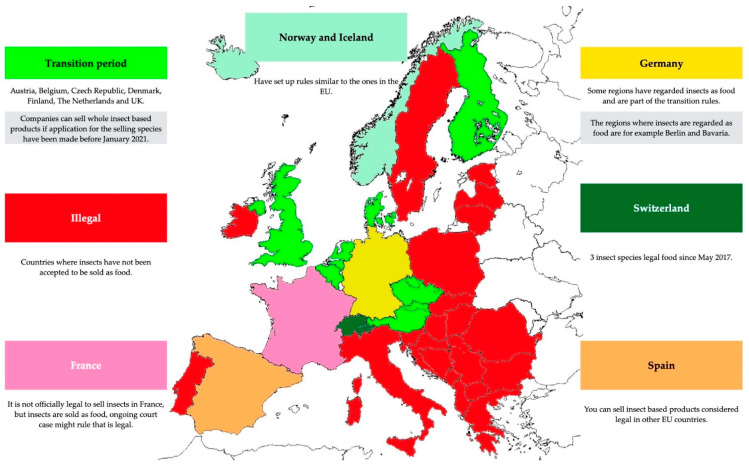
Insect Food Status in Europe. Adapted from Source: www.bugburger.se (accessed on 17 August 2021) [153].

An interesting fact is that insects were already being sold as food in the EU, but there had been doubts among the Member States on whether whole insects were covered by previous Novel Food Regulation. The uncertainty was clarified by the European Court of Justice in October 2020, which concluded that the whole insects were not in the scope of previous regulation [78]. After contacting the companies in Europe, it was also clear that the legislation within each country in Europe can differ (see Figure 3) and that companies are obliged to follow their country’s legislation [144,145,146,147,148,149] and some have not given their approval for mealworms for human consumption, e.g., in France [144]. However, insect protein was approved in September 2021 for poultry and pig feed [142] in Europe according to regulation no. 1372/2021 [142].

Different rules apply in European countries, especially regarding the transition period. The main change from 2019 is that in 2021 Tenebrio molitor was allowed for human consumption.

### 4.3. Sustainable Development Goals (SDG) and European Strategies

SDG are goals that were first set in 2015 by the international community to pledge countries of the world to eradicate poverty, find sustainable and inclusive developmental solutions and ensure everyone’s human right. There are 17 SDGs to be reached by 2030. These SDGs are: no poverty (SDG 1), zero hunger (SDG 2), good health and well-being (SDG 3), quality education (SDG 4), gender equality (SDG 5), clean water and sanitation (SDG 6), affordable and clean energy (SDG 7), decent work and economic growth (SDG 8), innovation, and infrastructure (SDG 9), reduced inequality (SDG 10), sustainable cities and communities (SDG 11), responsible consumption (SDG 12), climate action (SDG 13), life below water (SDG 14), life on land (SDG 15), peace, justice, and strong intuitions (SDG 16) and partnerships for the goals (SDG 17). The EU is committed to implement the SDGs in all of its policies and to encourage EU countries to do the same [154]. Insect rearing shows great potential to work towards the SDGs as it increases food security (SDG 2), improves waste management (SDG 12), and can have positive effects on human health and well-being (SDG 3). Furthermore, insect rearing with standardized techniques on an industrial scale is a novel economic sector able to improve the sustainability of the global food chain (SDG 9) [154].

The EU’s strategies are developed and translated into policies and initiatives by the European Commission. The European Commission has set 6 priorities for 2019–2024; one of these priorities is A European Green Deal (EGD) [155]. The EGD aims to “transform the EU into a fair and prosperous society, with modern, resource-efficient and competitive economy where there are no net emissions of greenhouse gases in 2050 and where economic growth is decoupled from resource use”. One of the strategies to reach the EGD goal is the Farm to Fork strategy, which was established to design a fair, healthy and environmentally friendly food system that has a global standard in sustainability and will contribute to achieving a circular economy [156]. The approval of mealworms as a novel food contributes to the objectives of the Green Deal and the Farm to Fork strategy [77].

### 4.4. Companies in Europe That Farm and Sell Insects

There exist several professional insect farming companies in Europe in various countries that farm mealworms and/or black soldier flies for either human or animal consumption, and some companies sell larva residues as fertilizer. In addition to mealworms and BSF, there are also some companies in Europe that farm other insect species, such as crickets [157]. Currently, the most used platform for the marketing of edible insect products are through companies’ own websites, followed by fairs/events/conferences [4]. In March 2017, it was estimated that more than 200 start-up insect farming companies existed in Europe [158]. These start-up companies often consist of unique characteristics like the way they are organized, the growth plan or the financing structure. In spite of this, over 60% of start-up companies go bankrupt within 5 years [139]. Bug Burger lists 68 insect start-ups in Europe that have disappeared for various reasons, one of them being bankruptcy [157].

**Figure 4:**
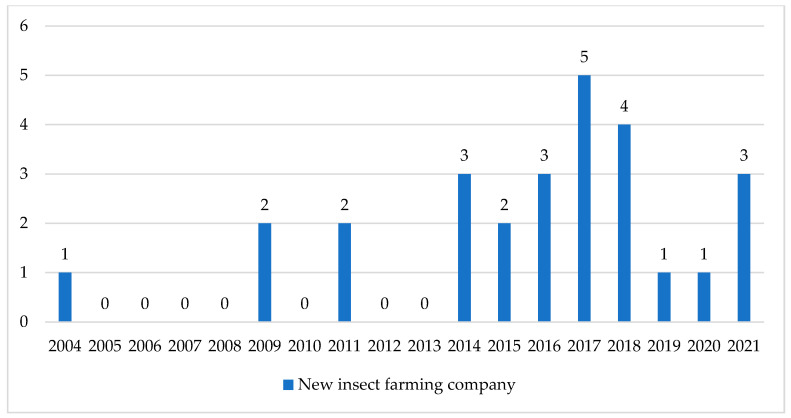
Foundation years of the 27 companies listed in Table 3.

**Figure 5:**
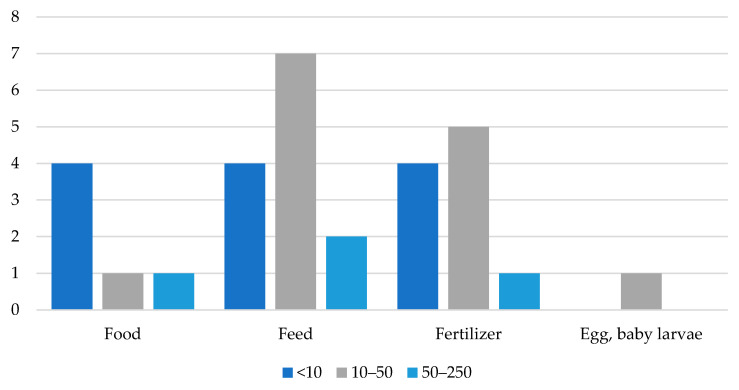
Number of employees based on the type of industry in the companies listed in Table 3.

For this article, 27 insect-farming companies in Europe that farm mealworms and/or BSF larvae were contacted and 9 have answered [144,145,146,147,148,149,159,160,161]. However, 3 out of 9 answered companies did not provide proper answers, one was closed due to COVID-19 [159], one does not drive with insect rearing anymore [160] and one had no time to answer [161]. In Table 3, these 27 companies are compared; the companies that gave answers are yellow while the companies that did not provide answers are white. The data from the companies that did not answer or did not give proper answers was provided through the companies own websites along with newspaper articles about these companies and LinkedIn. When researching these companies, it can be estimated that there are equally as many companies in Europe that breed mealworms as there are that breed BSF larvae. It can also be estimated that most insect farming companies in Europe were founded between 2014 and 2018, as seen in Figure 4. Most insect farming companies investigated for this article were farming insects as feed mostly for either pets or aquaculture; this is in accordance with the IPIFF website. According to a survey performed in 2020 by IPIFF on the EU market in March 2020, most companies in Europe that sell insects for food are micro companies or 81%, which means that they have below 10 employees and only 3% are considered medium-sized companies with 50–250 employees [4]. However, according to the IPIFF survey on the market of insect as feed in 2021, over 40% of all insect farming companies that sell insects as feed were micro companies in 2020 and over 20% were medium-sized companies [124]. According to the companies that were investigated for this article, 42% were micro [146,149,162,163,164,165,166,167], 60% were small [145,148,168,169,170,171,172,173,174] and 11% were medium-sized companies [144,175]. Figure 5 shows the size of the companies researched for this article based on the type of industry. Ÿnsect in France is the biggest insect breeding company in Europe. It was founded in 2011 and has around 230 employees today. Currently, in 2021, Ÿnsect is building what will be the world’s largest insect breeding facility and recently it also acquired an international branch in The Netherlands (Protifarm) [144].

**Figure 6:**
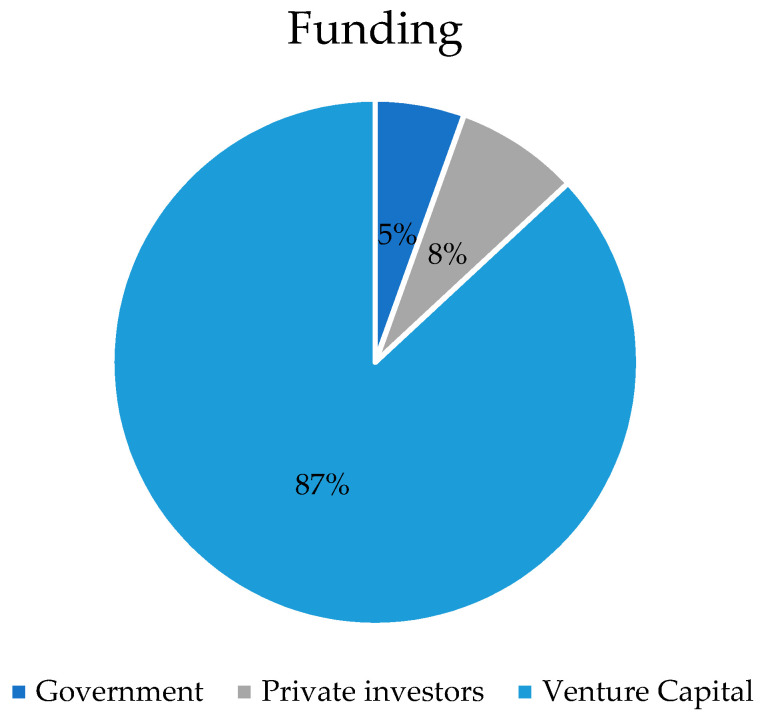
The prevalence of amount of investment in Ynsect, Nasekomo and Entomobio by the type of funding.

The total investment into the majority of insect farming companies in Europe is below 500 K euros (€), around 30% of companies get between, 1 to 5 million €, with 6% getting over 10 million € [4]. As seen in Figure 6, insect farming companies in Europe investigated for this article were mostly been funded by Venture Capital. Most of these European insect farming companies are not economically sustainable today and are dependent on funding. However, both Ÿnsect in France [144] and Nasekomo in Bulgaria are estimated to become fully sustainable in 2022 [148].

Of the companies which answered, 50% were producing insects with automatic methods [144,145,148] and the other half with manual methods [146,147,149]. Automatic methods are considered beneficial on the grounds of increasing productivity, efficiency and consistency and of decreasing human labour [176].

**Table 3:** 27 insect farming companies compared, yellow companies have given direct answers and the information from the white companies was provided through companies’ website, articles, and LinkedIn.

Company	Species	Type of Farm	Location	Foundation	Annual Revenue €	Economic Sustainability	Use	Funding €	Number of Employees	Member of IPIFF
Ÿnsect [144]	Mealworm	Automated	France, The Netherlands, and USA	2011	89 M	Estimated to be profitable in 2022	Aquaculture, domestic animals, fertilizer, for human consume soon	360 M	230 will be close to 400 in 2022	Yes
Hexafly [145]	BSF	Automated,	Ireland	2016	200 tons meal production annually		Feed for aquaculture, pets, and animals and fertilizer	Equity funded	30	Yes
Verteco Farm [146]	Mealworm	Manual	Sweden	2020	Negative		Fertilizer	None	2	No
Syklus [147]	BSF	Manual	The Netherlands	2021	12 tons larva per year	Not yet	For ornamental fish	Yes		No
Nasekomo [148]	BSF	Automated	Bulgaria	2017	150 K	Not yet estimated to be sustainable in 2022	Aquaculture, fertilizer, pets	5 M	42	Yes
Entomobio [149]	Mealworm		Belgium	2018	7 K in 2020	Not yet	Human	60 K	1–3 depending on the period	No
Cricky [159,177]	Mealworm and crickets	Closed due to COVID-19	Croatia	2016		Closed due to COVID-19	Human			No
Urbanmat [160,178]	Mealworm, BSF and other species	Not rearing anymore, purchase and sale of insect and storage	Norway	2017			Human			No
Insectum [161,179]	BSF		Denmark	2018			Feed			No
Horizon Edible Insects [162,180]	Mealworm	manual	UK	2019			Human	Guided tours and cooking classes	1–10	No
Tebrito [163,181]	Mealworm	manual	Sweden	2016			Human, frass		1–10	Yes
Entobreed [182]	Mealworm		The Netherlands	April 2021, first egg arrived, have not started selling			Fertilizer, feed, human	130 K Crowdfunding		No
Marienlyst Ento [183]	Mealworm	Manual	Denmark	2017			Human mostly to companies but also for private use			No
Micronutris [172,184]	Mealworm		France	2011			Human		11–50	No
Entoinnov [164,185]	Mealworm		France	2021			Human, feed, fertilizer		1–10	No
Entocycle [173,186]	Black solder fly		UK	2014			Pets, frass	Governmental backing	11–50	Yes
Protix [175]	Mealworm, BSF, grasshopper, crickets	Automated	The Netherlands, active in 12 countries	2009			Pets, feed, food, and fertilizer	>45 M several private investors	50–250	Yes
Enorm [165,187]	BSF	Automated	Denmark	2018	The goal is to produce 1.5 ton of living larva every day		Feed		1–10	Yes
Tebrio [174,188]	Mealworm	Automated	Spain	2014			Feed, pets, fertilizer, antibac, human in the future	Venture capital, government 40% interest	11–50	Yes
Bugimine [189]	Mealworm		Estonia	2017			Pets, aquaculture, poultry, fertilizer	Private capital		No
NextAlim [168]	BSF	Automated	France, plan to construct 3 additional product units in Europe and outside of Europe in 2022–2025	2014	2.4 tons of egg per year, estimated to be 12 tons of egg per year in 2022		Egg, neonates, and 5–7 days old larva ready to rear, to another companies	Yes	20	Yes
nextProtein [169]	BSF	Manual	France	2015			Pets, aquaculture, feed for other animals, fertilizer		15	Yes
Hermetia [166,190]	BSF	Manual	Germany	2009			Aquaculture		1–10	Yes
Illucens Gmbh [170]	BSF		Germany	2018			Pets and zoo animals		26	Yes
HiProMine [171]	BSF	Vertical	Poland	2015			Pets, aquaculture, feed for other animals in the future		15	Yes
EntoMass [167,191]	BSF	Manual	Denmark	2017			Pets, fertilizer		1–10	No
PAPEK s.r.o. [192]	Mealworm	Manual	The Czech Republic	2004			Export and zoos			

### 4.5. Consumer Acceptance

Consumer acceptance of entomophagy is important to start a large scale production of insects used for human food and it still remains the biggest challenge for the insect industry today [133]. In recent years, there has been a lot of research regarding the consumer’s acceptance of entomophagy in Europe, and over 200 scientific papers about this topic have been written. This article focuses on 10 research articles [130,131,132,133,134,135,193,194,195,196] on consumers acceptance of entomophagy in Europe and the results from one review article [130] that focuses on other 38 research articles of the same topic. Most research regarding the consumer acceptance of entomophagy in Europe has focused on consumers from Italy, the Netherlands and Belgium. Few articles have focused on consumer acceptance in Germany, Switzerland, Finland, Denmark, Czech Republic, Poland, France, Hungary, Sweden, and Ireland [129]. These studies show an increased interest in commercializing insect-based foods [132], e.g., the Netherlands published the first article about consumers acceptance of insects in 2012 from a survey performed in 2010. The results showed a low acceptability towards entomophagy in Dutch consumers [130]. In 2020, another study on consumer acceptance among Dutch and German students was performed. The results showed a higher acceptability towards insects, as food by the Dutch students rather than in the study performed in 2012 [131]. However, the participants in the 2020 study were on average younger than the participants in the 2012 study [130,131], but studies suggest that younger people are generally more willing to consume insects than the older generation [129,133]. Despite this increased interest in entomophagy, the number of studies indicate that high proportions of Europeans still consider insects as a food to be taboo [132] and many do not know that insects are consumed in Europe [133]. Consumers in Northern Europe seem to be more accepting towards entomophagy than consumers in Central, Mediterranean and Western Europe [129,194]. According to several studies, men seem to be more accepting towards edible insects than women [133], while other studies reported no difference [132]. As these research articles seem to show increased consumer acceptance with time, it is highly likely that the consumer acceptance towards entomophagy will continue to increase in the near future, especially as younger participants seem to show more acceptance towards edible insects than older ones. In Bangkok, it seems that the young people are among the main drivers where significant revival of insect-eating is happening, along with increasing interest from tourists. The prices there are getting higher, but even so, people are buying them and the market for edible insects is growing [8]. Therefore, consumer acceptance will likely remain the biggest challenge for insect farming development into more financially viable businesses for the next five years, as many Europeans still regard insects as a taboo food.

Several explanations are considered regarding the negative attitude of Europeans towards entomophagy. One of these explanations is food neophobia, which is defined as the unwillingness to try new foods [132,133] and is related to human innate paradoxical behaviour towards unknown or unfamiliar food and consider it to be a potential threat to their organism [132]. Consumer’s food neophobia tendencies have been shown to reduce the consumers’ willingness to eat insects both as a whole and as an ingredient in food [131,133,193,194]. However, food neophobia seems to be an extremely complex attitude and can vary during the course of one’s life [193]. It has also been stated that food neophobia is not as significant a barrier to insect consumption as it once it was, since edible insects are becoming more familiar to consumers [131].

Another explanation is disgust, but Europeans generally consider insects to be dirty [133], and view insects to be a pathogenic risk; therefore, food containing insects are considered disgusting [193] and repulsive [134]. Studies have shown that the feeling of disgust affects the willingness to consume insects negatively [133,134,193] and the feeling of disgust strongly influences perception, even before insect products are tasted [133]. The feeling of disgust is a complex phenomenon that could be associated with health risks posed by the consumption of a specific substance [134]. Disgust toward a specific food generally comes from culturally induced rejection [133,134]. It is conceptualized as an adaptive reaction and is closely connected to the information people have at the time. People can change their food preference through information, exchange, and experience [134]. According to several studies, food neophobia, along with disgust, have the most negative influence on acceptance of insect products [133].

A third explanation is insect phobia, but a study done by Moruzzo et al. [193] has shown that insect phobia has a more negative influence on the willingness towards tasting insects than food neophobia. In the future, when insects will be a more known food in Europe, insect phobia will have more of an effect than food neophobia on the intention to eat food containing insects and an increasing familiarity with insect food will not be enough for consumers to adopt insect-based food.

According to Meyer-Rochow et al. [195], Europeans attitudes towards edible insects might be influenced by idioms containing unfavourable references to insects. Idioms occur in all languages and can have an important influence on society and become integrated into feelings like irritation, contemptuous attitude, anger, and disgust. Idioms that exist in European languages convey predominantly negative attitudes, while the opposite is true in East Asia. Mirror neurons are believed to be activated when listening to idioms and could lead the listener of idioms towards a negative attitude towards insects and project it towards edible species. New idioms appear all the time and perhaps making more positive idioms towards insects might help change attitudes.

To increase consumer acceptance, persuasion tactics to reduce Europeans’ anxiety towards entomophagy is important. These persuasion tactics can help to disguise insects in food, combining them with familiar ingredients or turning them into powder [132], but many studies have reported a higher consumer acceptance towards processed insect products rather than to whole insects [130,133,134,135,196]. Other tactics are to increase the familiarity with insects as a food by having them in grocery stores and talking to friends that have a positive experience with edible insects [132]. Increasing the knowledge about the positive environmental effects regarding edible insects and their health benefits has also been proven to improve consumer attitude towards entomophagy [131,132,133,134,135]. Even though insects are considered a taboo food today, this attitude might change. Sushi was once considered to be a taboo food, but it has increased in popularity in the recent years [132].

## 5. Iceland

Iceland has abundant resources, e.g., land, water and renewable energy (geothermal and hydro power). As an island in the North Atlantic Ocean, it is important to be sustainable in terms of food and feed. Today, the country imports fuel, fertilizer, feed raw materials, feed and food. Insects could be a valuable factor in supporting food security in Iceland and could serve an important role in food circulation.

### 5.1. Insect Trials in Recent Years

Limited research has been conducted regarding insect breeding and the use of insects as food and feed in Iceland. In 2014, black soldier flies were experimentally bred as a potential feed ingredient for fish farming in northwest Iceland, but the activity was discontinued. There were several reasons for this activity being discontinued. One of these reasons was that the EU laws regarding insect farming were under construction, and it seemed unlikely that the EU would allow the ideas on which the company based its business [197]. Moreover, according to the owners of the company, there was not enough underutilized food in Iceland to make it a stable feed for black soldier flies [198].

Another experiment in Iceland started in 2015 with the production of protein bars from crickets (Jungle Bar). The production went well, as well as the marketing, and the idea seemed to be well received by Icelanders [199]. However, only a few days after the product was launched in the stores the Icelandic government implemented European law prohibiting the sale of insect-based products for human consumption and the project had to be discontinued [200].

In 2018, a new start-up on breeding mealworms received funding. The plan was to produce mealworms for aquaculture and breeding the insects, at stable conditions with the use of geothermal heat [201]. However, the project was discontinued beyond 2019.

Additionally, BSF were experimentally bred in the governmentally owned, food and biotech R&D company in Iceland, Matís, from 2012 to 2014. One of the aims of the study was to examine the effect of different organic waste on the nutritional content of the larvae. Matís presented its findings in the international conference, Insect to feed the World, in 2014. The results showed that it is possible to have a great influence on the nutritional content of the larvae with different feeds [202]. In 2019, a new study relating to insect proteins was started in Matís. This project is estimated to take 4 years and it is about breeding crickets for the larvae to be used in bread [203]. Currently, crickets are not authorized for human consumption; however, in august 2021, The European Food and Safety Agency (EFSA) submitted an option on the safety of frozen and dried formulations from whole house crickets (*Acheta domesticus*) as a novel food pursuant to the European Commission, and the authorisation can be expected in early 2022 [136].

Iceland is also a part of the pan-European project NextGenProteins, which is optimizing the production of three alternative protein sources, with one of them deriving from black soldier flies and crickets [203].

Currently, mealworms and BSF are experimentally bred in the Agricultural University of Iceland. The mealworms are being fed with waste from Icelandic brewery production and carrots and the BSF larvae are being fed with kitchen waste [204].

As of today, no study of consumer acceptance of edible insects has been performed on Icelandic consumers. It is not unlikely that consumer acceptance in Iceland resembles the consumers acceptance in Europe mentioned in Section 4.3.

### 5.2. The Laws and Regulations

According to EU regulation no. 2015/2283, whole insects are in scope as well as parts of whole insects, powder and extracts. Insects cannot yet be fed with feed ingredients that are not authorized for farmed animals [205]. When the EU regulation 2015/2283 was implemented, the startup company Crowbar Protein in Iceland was working on the development of the previously mentioned Jungle Bar. The product, launched in January 2016, was requested to be pulled off the shelves, although the current regulation no. 2015/2283 was entered into force in Iceland 1st of January 2018 [206]. After only one week, the Jungle Bar was pulled off the shelves, requested by the Icelandic authorities referring to EU regulations. One of the owners of the startup company reported that they had submitted all documentation to the Directorate of Health and the Icelandic Food and Veterinary Authority to confirm that the products were safe for human consumption. The owners of Crowbar Protein subsequently signed a contract with a distributor in the USA [207]. Another company said at that time that their hands were tied by uncertain regulations [208].

Since then, new regulations have emerged in Europe but, as explained in Section 4.1, different countries have quite different rules. According to the Icelandic Food and Veterinary Authority, Iceland follows the regulations from the EU and evaluations from the EFSA. Therefore, it is now allowed to start marketing yellow mealworms in Iceland (Commission Implementing Regulation (EU) 2021/882 from 1st of June 2021) [206].

### 5.3. Importing Feed

Iceland imports feed, e.g., soybean meal and grain, especially for aquaculture, poultry and pigs, but based on work on food security, several opportunities for increased production in Iceland have been identified [209]. It is possible to make use of natural resources, become more sustainable, improve food security, e.g., if the import is uncertain as in pandemic times, use less currency, and create more jobs and use knowledge in Iceland. One goal could be establishing insect farming, but there is also exciting research going on with different grain cultivation in Iceland.

With insect farming, waste from agriculture as well as the essential food waste within the country and even preferentially from the next neighborhood could be used. By doing this, a sustainable cycle and food security is better maintained.

### 5.4. Food Waste in Iceland

According to the Food and Agriculture Organization of the United Nations (FAO), more than one third of food is wasted and at all stages of production. This waste contributes significantly to greenhouse gasses and climate change. At the same time, hunger exists in the world and the population has been growing 7-fold over the last 200 years and is still growing. According to the FAO (2018), a 60% increase will be needed to meet growing demands of the world population. Food waste is very complex and interdisciplinary research teams are starting to use model-driven integrative applied research approaches. The Recovery Food Hierarchy (EPA, 2019, appendix A) constitutes these main headlines: (1) Source Reduction; (2) Food for people; (3) Feed for animals; (4) Industrial Usage; (5) Composting; and at last (6), Landfill incineration [210].

Another primary idea is increased awareness, e.g., children who can participate in making the food they eat are less likely to throw it away. According to Icelandic research from 2016, it is estimated that the food waste is 23 kg of food that could have been used and 35 kg of food defined as non-useable (e.g., eggshell, coffee basket, bones and peel of vegetables and fruits) per individual in Iceland. The Icelandic information site *matarsoun.is* is a project led by The Environment Agency of Iceland (Umhverfisstofnun). There it is stated that the worth of the food wasted was 4.5 billion ISK in 2015 [211].

## 6. Conclusions

In recent years, the interest in insect farming has been increasing in Europe due to the need for new food sources with less of an environmental impact than conventional production. The United Nations identifies insects as a food product that could increase food security and human health and could reduce pollution, and the European Union supports innovation and research in these fields.

Studies have shown that the nutritional profile of both mealworms and BSF larvae is good for human consumption, as feed for animals, being high in protein, fat, fiber and several vitamins and minerals. Furthermore, studies have shown that eating insects might reduce cholesterol levels in the body. Insects are not only nutritious, but they also contain substances that could promote the immune system. Using insects as animal feed could lead to a reduction in antibiotic use in livestock. Insect farming is more environmentally friendly than the farming of traditional livestock and the production of soybean and fishmeal as insect production uses less arable land and water and results in lesser greenhouse gas emissions. Furthermore, studies have shown that insects, e.g., mealworms and BSF larvae, can be used to degrade several types of organic waste and that mealworms can be used to degrade plastics. However, the true environmental impact of large-scale insect farming is unknown, and more studies could focus on that.

For novel products such as food and feed to be placed on the European market, the product must be safe. Several studies have focused on the safety of edible insects and the safety risks that have been identified are allergies, toxins, and zoonotic pathogens. Studies have shown that insect protein has a cross reactivity with crustacea and mite allergies, concluding that people who suffer from these allergies should not consume or work around edible insects. Toxicity studies of mealworms from the literature do not raise safety concerns according to the EFSA and yellow mealworm has now been authorized for the market as a novel food product. More applications for other species are pending. It is interesting that different countries in Europe have different rules, but it is important to evaluate risk profiles. When hygiene and other safety needs are met, it seems that the risk profiles are like other products. To finish the feed and food circulation, it would be important to use other food waste. Research is also ongoing into how insects manage with plastics. As insects are so phylogenetically different from humans and other mammals, studies indicate that edible insects pose a smaller risk to humans than traditional livestock. However, some pathogenic bacteria, viruses and parasites have been identified in edible insect farms in Europe. Microbiota can be introduced during farming and processing and therefore good hygiene strategies should be on place on the farm. Additionally, the substrate used to feed the insects majorly affects the insect’s microbiota and composition; therefore, it is important to choose the substrate well and to store it under proper conditions.

Interest in insect farming in Europe is growing and the biggest European company in this sector has approximately 230 employees. Regulations are developing, and investors and competition funds are supporting the development. Most importantly, European consumers are becoming more positive both toward insects as animal feed and as food for human consumption.

## Data Availability

Data is contained within the article.

## References

[1] Evans J., Alemu M., Flore R., Frøst M., Halloran A., Jensen A., Maciel G., Meyer-Rochow V., Münke-Svendsen C., Olsen S. (2015). ‘Entomophagy’: An evolving terminology in need of review. J. Insects Food Feed.

[2] Raheem D., Carrascosa C., Oluwole O.B., Nieuwland M., Saraiva A., Millán R., Raposo A. (2019). Traditional consumption of and rearing edible insects in Africa, Asia and Europe. Crit. Rev. Food Sci. Nutr..

[3] Bordiean A., Krzyżaniak M., Stolarski M.J., Czachorowski S., Peni D. (2020). Will Yellow Mealworm Become a Source of Safe Proteins for Europe?. Agriculture.

[4] International Platform of Insects for Food and Feed (2020). Edible Insects on the European Market.

[5] Worldometer Europe Population (LIVE). https://www.worldometers.info/world-population/europe-population/.

[6] Huis A., Van Itterbeeck J., Klunder H., Mertens E., Halloran A., Muir G., Vantomme P. (2013). Edible Insects Future Prospects fo Food and Feed Security.

[7] Worldometer World Population. https://www.worldometers.info.

[8] Müller A. (2019). Insects as Food in Laos and Thailand. Asian J. Soc. Sci..

[9] De Carvalho N.M., Madureira A.R., Pintado M.E. (2020). The potential of insects as food sources—A review. Crit. Rev. Food Sci. Nutr..

[10] Van Huis A. (2016). Edible insects are the future?. Proc. Nutr. Soc..

[11] Kiple K.F. (2007). A Movable Feast: Ten Millennia of Food Globalization.

[12] Massard J.A. (2007). Maikäfer in Luxemburg: Historisches und Kurioses. Lëtzebuerger J..

[13] Meyer-Rochow V. (1975). Can insects help to ease the problem of world food shortage?. Search.

[14] United Nations Discussion Starter Action Track 3: Boost Nature-Positive Food Production at Scale. https://www.un.org/sites/un2.un.org/files/unfss-at3-discussion_starter-dec2020.pdf.

[15] Food and Agriculture Organization, International Fund for Agricultural Development, United Nations Children’s Fund, World Health Organization (2021). The State of Food Security and Nutrition in the World 2021: Transforming Food Systems for Food Security, Improved Nutrition and Affordable Healthy Diets for All.

[16] Hodson E., Niggli U., Kaoru K., Lal R., Sadoff C. Boost Nature Positive Production at Sufficient Scale—A Paper on Action Track 3. Proceedings of the United Nations Food Systems Summit 2021.

[17] Grau T., Vilcinskas A., Joop G. (2017). Sustainable farming of the mealworm Tenebrio molitor for the production of food and feed. Z. Für Nat. C.

[18] Hong J., Han T., Kim Y.Y. (2020). Mealworm (*Tenebrio molitor* Larvae) as an Alternative Protein Source for Monogastric Animal: A Review. Animals.

[19] Dortmans B., Diener S., Verstappen B., Zurbrügg C. (2017). Black Soldier Fly Biowaste Processing—A Step-by-Step Guide.

[20] Sharma R., Kaur R., Rana N., Poonia A., Rana D.C., Attri S. (2021). Termite’s potential in solid waste management in Himachal Pradesh: A mini review. Waste Manag. Res..

[21] Thévenot A., Rivera J.L., Wilfart A., Maillard F., Hassouna M., Senga-Kiesse T., Le Féon S., Aubin J. (2018). Mealworm meal for animal feed: Environmental assessment and sensitivity analysis to guide future prospects. J. Clean. Prod..

[22] The Donau Soja Association Soya Bean History. https://www.donausoja.org/en/research/agriculture/soya-bean-history/.

[23] European Fishmeal and Fish Oil Producers We Represent the European Fishmeal and Fish Oil Producers in Denmark, Faroe Islands, Iceland, Ireland, Norway, United Kingdom, Estonia and Spain. https://effop.org.

[24] Belghit I., Liland N.S., Waagbø R., Biancarosa I., Pelusio N., Li Y., Krogdahl Å., Lock E.-J. (2018). Potential of insect-based diets for Atlantic salmon (*Salmo salar*). Aquaculture.

[25] Selaledi L., Hassan Z., Manyelo T.G., Mabelebele M. (2021). Insects’ Production, Consumption, Policy, and Sustainability: What Have We Learned from the Indigenous Knowledge Systems?. Insects.

[26] Finke M.D. (2015). Complete nutrient content of four species of commercially available feeder insects fed enhanced diets during growth. Zoo Biol..

[27] Nyangena D., Mutungi C., Imathiu S., Kinyuru J., Affognon H., Ekesi S., Nakimbugwe D., Fiaboe K. (2020). Effects of Traditional Processing Techniques on the Nutritional and Microbiological Quality of Four Edible Insect Species Used for Food and Feed in East Africa. Foods.

[28] Hermans W.J.H., Senden J.M., Churchward-Venne T.A., Paulussen K.J.M., Fuchs C.J., Smeets J.S.J., van Loon J.J.A., Verdijk L.B., van Loon L.J.C. (2021). Insects are a viable protein source for human consumption: From insect protein digestion to postprandial muscle protein synthesis in vivo in humans: A double-blind randomized trial. Am. J. Clin. Nutr..

[29] Gessner D.K., Schwarz A., Meyer S., Wen G., Most E., Zorn H., Ringseis R., Eder K. (2019). Insect Meal as Alternative Protein Source Exerts Pronounced Lipid-Lowering Effects in Hyperlipidemic Obese Zucker Rats. J. Nutr..

[30] Kim T.K., Yong H.I., Kim Y.B., Kim H.W., Choi Y.S. (2019). Edible Insects as a Protein Source: A Review of Public Perception, Processing Technology, and Research Trends. Food Sci. Anim. Resour..

[31] Mariod A.A., Adam Mariod A. (2020). Nutrient Composition of Mealworm (Tenebrio Molitor). African Edible Insects as Alternative Source of Food, Oil, Protein and Bioactive Components.

[32] Liu X., Chen X., Wang H., Yang Q., Ur Rehman K., Li W., Cai M., Li Q., Mazza L., Zhang J. (2017). Dynamic changes of nutrient composition throughout the entire life cycle of black soldier fly. PLoS ONE.

[33] EFSA Panel on Nutrition N.F., Allergens F., Turck D., Castenmiller J., De Henauw S., Hirsch-Ernst K.I., Kearney J., Maciuk A., Mangelsdorf I., McArdle H.J. (2021). Safety of dried yellow mealworm (*Tenebrio molitor* larva) as a novel food pursuant to Regulation (EU) 2015/2283. EFSA J..

[34] Kouřimská L., Adámková A. (2016). Nutritional and sensory quality of edible insects. NFS J..

[35] Oonincx D.G.A.B., Laurent S., Veenenbos M.E., van Loon J.J.A. (2020). Dietary enrichment of edible insects with omega 3 fatty acids. Insect Sci..

[36] Payne C.L., Scarborough P., Rayner M., Nonaka K. (2016). Are edible insects more or less ‘healthy’ than commonly consumed meats? A comparison using two nutrient profiling models developed to combat over- and undernutrition. Eur. J. Clin. Nutr..

[37] Baek M., Kim M.A., Yun-Suk K., Hwang J.S., Goo T.-W., Jun M., Yun E.-Y. (2019). Effects of processing methods on nutritional composition and antioxidant activity of mealworm (*Tenebrio molitor*) larvae. Entomol. Res..

[38] Wendin K., Mårtensson L., Djerf H., Langton M. (2020). Product Quality during the Storage of Foods with Insects as an Ingredient: Impact of Particle Size, Antioxidant, Oil Content and Salt Content. Foods.

[39] Mouithys-Mickalad A., Schmitt E., Dalim M., Franck T., Tome N.M., van Spankeren M., Serteyn D., Paul A. (2020). Black Soldier Fly (*Hermetia illucens*) Larvae Protein Derivatives: Potential to Promote Animal Health. Animals.

[40] Rawski M., Mazurkiewicz J., Kierończyk B., Józefiak D. (2020). Black Soldier Fly Full-Fat Larvae Meal as an Alternative to Fish Meal and Fish Oil in Siberian Sturgeon Nutrition: The Effects on Physical Properties of the Feed, Animal Growth Performance, and Feed Acceptance and Utilization. Animals.

[41] Liland N.S., Biancarosa I., Araujo P., Biemans D., Bruckner C.G., Waagbø R., Torstensen B.E., Lock E.J. (2017). Modulation of nutrient composition of black soldier fly (*Hermetia illucens*) larvae by feeding seaweed-enriched media. PLoS ONE.

[42] Tschirner M., Simon A. (2015). Influence of different growing substrates and processing on the nutrient composition of black soldier fly larvae destined for animal feed. J. Insects Food Feed.

[43] USDA Database. Release 28. https://www.ars.usda.gov.

[44] Office of Nutrition, Labeling and Dietary Supplements in the Center for Foood Safety and Applied Nutrition at the US Food and Drug Administration (2013). A Food Labeling Guide: Guidance for Industry.

[45] Alves A., Sanjinez-Argandoña E., Linzmeier A., Cardoso C., Macedo L. (2016). Food Value of Mealworm Grown on Acrocomia aculeata Pulp Flour. PLoS ONE.

[46] Kawasaki K., Hashimoto Y., Hori A., Kawasaki T., Hirayasu H., Iwase S.I., Hashizume A., Ido A., Miura C., Miura T. (2019). Evaluation of Black Soldier Fly (*Hermetia illucens*) Larvae and Pre-Pupae Raised on Household Organic Waste, as Potential Ingredients for Poultry Feed. Animals.

[47] Ravzanaadii N., Kim S., Choi W.H., Hong S.-J., Kim N. (2012). Nutritional Value of Mealworm, Tenebrio molitor as Food Source. Int. J. Ind. Entomol..

[48] Schmitt E., Belghit I., Johansen J., Leushuis R., Lock E.J., Melsen D., Ramasamy Shanmugam R.K., Van Loon J., Paul A. (2019). Growth and Safety Assessment of Feed Streams for Black Soldier Fly Larvae: A Case Study with Aquaculture Sludge. Animals.

[49] Benzertiha A., Kierończyk B., Rawski M., Józefiak A., Kozłowski K., Jankowski J., Józefiak D. (2019). Tenebrio molitor and Zophobas morio Full-Fat Meals in Broiler Chicken Diets: Effects on Nutrients Digestibility, Digestive Enzyme Activities, and Cecal Microbiome. Animals.

[50] Sogari G., Amato M., Biasato I., Chiesa S., Gasco L. (2019). The Potential Role of Insects as Feed: A Multi-Perspective Review. Animals.

[51] Islam M.M., Yang C.J. (2017). Efficacy of mealworm and super mealworm larvae probiotics as an alternative to antibiotics challenged orally with Salmonella and E. coli infection in broiler chicks. Poult. Sci..

[52] Primex Iceland. http://www.primex.is.

[53] Złotko K., Waśko A., Kamiński D.M., Budziak-Wieczorek I., Bulak P., Bieganowski A. (2021). Isolation of Chitin from Black Soldier Fly (*Hermetia illucens*) and Its Usage to Metal Sorption. Polymers.

[54] Lopez-Santamarina A., Mondragon A.d.C., Lamas A., Miranda J.M., Franco C.M., Cepeda A. (2020). Animal-Origin Prebiotics Based on Chitin: An Alternative for the Future? A Critical Review. Foods.

[55] Oonincx D.G., van Itterbeeck J., Heetkamp M.J., van den Brand H., van Loon J.J., van Huis A. (2010). An exploration on greenhouse gas and ammonia production by insect species suitable for animal or human consumption. PLoS ONE.

[56] Halloran A., Roos N., Eilenberg J., Cerutti A., Bruun S. (2016). Life cycle assessment of edible insects for food protein: A review. Agron. Sustain. Dev..

[57] Berggren Å., Jansson A., Low M. (2019). Approaching Ecological Sustainability in the Emerging Insects-as-Food Industry. Trends Ecol. Evol..

[58] Smetana S., Schmitt E., Mathys A. (2019). Sustainable use of Hermetia illucens insect biomass for feed and food: Attributional and consequential life cycle assessment. Resour. Conserv. Recycl..

[59] Khan S., Khan R.U., Alam W., Sultan A. (2018). Evaluating the nutritive profile of three insect meals and their effects to replace soya bean in broiler diet. J. Anim. Physiol. Anim. Nutr..

[60] Motte C., Rios A., Lefebvre T., Do H., Henry M., Jintasataporn O. (2019). Replacing Fish Meal with Defatted Insect Meal (Yellow Mealworm *Tenebrio molitor*) Improves the Growth and Immunity of Pacific White Shrimp (*Litopenaeus vannamei*). Animals.

[61] Poore J., Nemecek T. (2018). Reducing food’s environmental impacts through producers and consumers. Science.

[62] Miglietta P.P., De Leo F., Ruberti M., Massari S. (2015). Mealworms for Food: A Water Footprint Perspective. Water.

[63] Alexander P., Brown C., Arneth A., Dias C., Finnigan J., Moran D., Rounsevell M.D.A. (2017). Could consumption of insects, cultured meat or imitation meat reduce global agricultural land use?. Glob. Food Secur..

[64] Oonincx D., Boer I.J.M. (2012). Environmental Impact of the Production of Mealworms as a Protein Source for Humans—A Life Cycle Assessment. PLoS ONE.

[65] Gligorescu A., Fischer C.H., Larsen P.F., Nørgaard J.V., Heckman L.-H.L. (2020). Production and Optimization of *Hermetia illucens* (L.) Larvae Reared on Food Waste and Utilized as Feed Ingredient. Sustainability.

[66] Bosch G., van Zanten H.H.E., Zamprogna A., Veenenbos M., Meijer N.P., van der Fels-Klerx H.J., van Loon J.J.A. (2019). Conversion of organic resources by black soldier fly larvae: Legislation, efficiency and environmental impact. J. Clean. Prod..

[67] Mekonnen M.M., Neale C.M.U., Ray C., Erickson G.E., Hoekstra A.Y. (2019). Water productivity in meat and milk production in the US from 1960 to 2016. Environ. Int..

[68] Alexander P., Brown C., Arneth A., Finnigan J., Rounsevell M.D.A. (2016). Human appropriation of land for food: The role of diet. Glob. Environ. Chang..

[69] Nielsen I., Jørgensen M., Bahrndorff S. (2011). Greenhouse Gas Emission from the Danish Broiler Production Estimated via LCA Methodology.

[70] Čičková H., Newton G.L., Lacy R.C., Kozánek M. (2015). The use of fly larvae for organic waste treatment. Waste Manag..

[71] Surendra K.C., Tomberlin J.K., van Huis A., Cammack J.A., Heckmann L.-H.L., Khanal S.K. (2020). Rethinking organic wastes bioconversion: Evaluating the potential of the black soldier fly (*Hermetia illucens* (L.)) (Diptera: Stratiomyidae) (BSF). Waste Manag..

[72] Tsochatzis E.D., Berggreen I.E., Nørgaard J.V., Theodoridis G., Dalsgaard T.K. (2021). Biodegradation of expanded polystyrene by mealworm larvae under different feeding strategies evaluated by metabolic profiling using GC-TOF-MS. Chemosphere.

[73] Hardy A., Benford D., Noteborn H., Halldorsson T., Schlatter J., Solecki R., Jeger M., Knutsen H., More S., Mortensen A. (2015). Risk profile related to production and consumption of insects as food and feed. EFSA J..

[74] Roháček J., Hora M. (2013). A northernmost European record of the alien black soldier fly *Hermetia illucens* (Linnaeus, 1758) (Diptera: Stratiomyidae). Časopis Slez. Zemského Muz.—Ser. A.

[75] Kooh P., Jury V., Laurent S., Audiat-Perrin F., Sanaa M., Tesson V., Federighi M., Boué G. (2020). Control of Biological Hazards in Insect Processing: Application of HACCP Method for Yellow Mealworm (*Tenebrio molitor*) Powders. Foods.

[76] Global Feed Safety Platform Why Is Feed Safety Important. http://www.fao.org/feed-safety/background/why-feed-safety/en/.

[77] European Commission Approval of First Insect as Novel Food. https://ec.europa.eu/food/safety/novel-food/authorisations/approval-first-insect-novel-food_en.

[78] Poma G., Yin S., Tang B., Fujii Y., Cuykx M., Covaci A. (2019). Occurrence of Selected Organic Contaminants in Edible Insects and Assessment of Their Chemical Safety. Environ. Health Perspect..

[79] Schrögel P., Wätjen W. (2019). Insects for Food and Feed-Safety Aspects Related to Mycotoxins and Metals. Foods.

[80] Huis A.V. (2020). Insects as food and feed, a new emerging agricultural sector: A review. J. Insects Food Feed.

[81] Petroski W., Minich D.M. (2020). Is There Such a Thing as “Anti-Nutrients”? A Narrative Review of Perceived Problematic Plant Compounds. Nutrients.

[82] Meyer-Rochow V.B., Gahukar R.T., Ghosh S., Jung C. (2021). Chemical Composition, Nutrient Quality and Acceptability of Edible Insects Are Affected by Species, Developmental Stage, Gender, Diet, and Processing Method. Foods.

[83] Stoops J., Crauwels S., Waud M., Claes J., Lievens B., Van Campenhout L. (2016). Microbial community assessment of mealworm larvae (*Tenebrio molitor*) and grasshoppers (*Locusta migratoria* migratorioides) sold for human consumption. Food Microbiol..

[84] Raimondi S., Spampinato G., Macavei L.I., Lugli L., Candeliere F., Rossi M., Maistrello L., Amaretti A. (2020). Effect of Rearing Temperature on Growth and Microbiota Composition of Hermetia illucens. Microorganisms.

[85] Caparros Megido R., Desmedt S., Blecker C., Béra F., Haubruge É., Alabi T., Francis F. (2017). Microbiological Load of Edible Insects Found in Belgium. Insects.

[86] Vandeweyer D., Crauwels S., Lievens B., Van Campenhout L. (2017). Microbial counts of mealworm larvae (*Tenebrio molitor*) and crickets (*Acheta domesticus* and *Gryllodes sigillatus*) from different rearing companies and different production batches. Int. J. Food Microbiol..

[87] Wynants E., Frooninckx L., Crauwels S., Verreth C., De Smet J., Sandrock C., Wohlfahrt J., Van Schelt J., Depraetere S., Lievens B. (2019). Assessing the Microbiota of Black Soldier Fly Larvae (*Hermetia illucens*) Reared on Organic Waste Streams on Four Different Locations at Laboratory and Large Scale. Microb. Ecol..

[88] Grabowski N.T., Klein G. (2017). Microbiology of processed edible insect products—Results of a preliminary survey. Int. J. Food Microbiol..

[89] Stastnik O., Novotny J., Roztocilova A., Kouril P., Kumbar V., Cernik J., Kalhotka L., Pavlata L., Lacina L., Mrkvicova E. (2021). Safety of Mealworm Meal in Layer Diets and their Influence on Gut Morphology. Animals.

[90] Tanga C.M., Waweru J.W., Tola Y.H., Onyoni A.A., Khamis F.M., Ekesi S., Paredes J.C. (2021). Organic Waste Substrates Induce Important Shifts in Gut Microbiota of Black Soldier Fly (*Hermetia illucens* L.): Coexistence of Conserved, Variable, and Potential Pathogenic Microbes. Front. Microbiol..

[91] Mancini S., Paci G., Ciardelli V., Turchi B., Pedonese F., Fratini F. (2019). Listeria monocytogenes contamination of Tenebrio molitor larvae rearing substrate: Preliminary evaluations. Food Microbiol..

[92] Swinscoe I., Oliver D.M., Ørnsrud R., Quilliam R.S. (2020). The microbial safety of seaweed as a feed component for black soldier fly (*Hermetia illucens*) larvae. Food Microbiol..

[93] Fasolato L., Cardazzo B., Carraro L., Fontana F., Novelli E., Balzan S. (2018). Edible processed insects from e-commerce: Food safety with a focus on the Bacillus cereus group. Food Microbiol..

[94] Garofalo C., Milanović V., Cardinali F., Aquilanti L., Clementi F., Osimani A. (2019). Current knowledge on the microbiota of edible insects intended for human consumption: A state-of-the-art review. Food Res. Int..

[95] Strother K.O., Steelman C.D., Gbur E.E. (2005). Reservoir competence of lesser mealworm (Coleoptera: Tenebrionidae) for Campylobacter jejuni (Campylobacterales: Campylobacteraceae). J. Med. Entomol..

[96] Van der Fels-Klerx H.J., Camenzuli L., Belluco S., Meijer N., Ricci A. (2018). Food Safety Issues Related to Uses of Insects for Feeds and Foods. Compr. Rev. Food Sci. Food Saf..

[97] Doi H., Gałęcki R., Mulia R.N. (2021). The merits of entomophagy in the post COVID-19 world. Trends Food Sci. Technol..

[98] Ochoa Sanabria C., Hogan N., Madder K., Gillott C., Blakley B., Reaney M., Beattie A., Buchanan F. (2019). Yellow Mealworm Larvae (*Tenebrio molitor*) Fed Mycotoxin-Contaminated Wheat-A Possible Safe, Sustainable Protein Source for Animal Feed?. Toxins.

[99] Bosch G., Fels-Klerx H.J.V., Rijk T.C., Oonincx D. (2017). Aflatoxin B1 Tolerance and Accumulation in Black Soldier Fly Larvae (*Hermetia illucens*) and Yellow Mealworms (*Tenebrio molitor*). Toxins.

[100] Camenzuli L., Van Dam R., De Rijk T., Andriessen R., Van Schelt J., Van der Fels-Klerx H.J. (2018). Tolerance and Excretion of the Mycotoxins Aflatoxin B1, Zearalenone, Deoxynivalenol, and Ochratoxin A by Alphitobius diaperinus and Hermetia illucens from Contaminated Substrates. Toxins.

[101] Schmidt G.D. (1971). Acanthocephalan infections of man, with two new records. J. Parasitol..

[102] Gałęcki R., Sokół R. (2019). A parasitological evaluation of edible insects and their role in the transmission of parasitic diseases to humans and animals. PLoS ONE.

[103] Percipalle M., Salvaggio A., Pitari G.M., Giunta R.P., Aparo A., Alfonzetti T., Marino A.M.F. (2021). Edible Insects and Toxoplasma gondii: Is It Something We Need To Be Concerned About?. J. Food Prot..

[104] Da Cruz C.E.F., Marques S.M.T., Andretta I. (2019). Endoparasites observed within invertebrates used as live food items for captive wild birds: Overview and potential risks. Zoo Biol..

[105] Müller A., Wiedmer S., Kurth M. (2019). Risk Evaluation of Passive Transmission of Animal Parasites by Feeding of Black Soldier Fly (*Hermetia illucens*) Larvae and Prepupae. J. Food Prot..

[106] Gałęcki R., Michalski M.M., Wierzchosławski K., Bakuła T. (2020). Gastric canthariasis caused by invasion of mealworm beetle larvae in weaned pigs in large-scale farming. BMC Vet. Res..

[107] Aelami M.H., Khoei A., Ghorbani H., Seilanian-Toosi F., Poustchi E., Hosseini-Farash B.R., Moghaddas E. (2019). Urinary Canthariasis Due to Tenebrio molitor Larva in a Ten-Year-Old Boy. J. Arthropod-Borne Dis..

[108] Ma J., Wang F. (2014). Prion disease and the ‘protein-only hypothesis’. Essays Biochem..

[109] Post K., Riesner D., Walldorf V., Mehlhorn H. (1999). Fly larvae and pupae as vectors for scrapie. Lancet.

[110] Thackray A.M., Muhammad F., Zhang C., Denyer M., Spiropoulos J., Crowther D.C., Bujdoso R. (2012). Prion-induced toxicity in PrP transgenic Drosophila. Exp. Mol. Pathol..

[111] Carp R.I., Meeker H.C., Rubenstein R., Sigurdarson S., Papini M., Kascsak R.J., Kozlowski P.B., Wisniewski H.M. (2000). Characteristics of scrapie isolates derived from hay mites. J. Neurovirol..

[112] Wisniewski H.M., Sigurdarson S., Rubenstein R., Kascsak R.J., Carp R.I. (1996). Mites as vectors for scrapie. Lancet.

[113] Raeber A.J., Muramoto T., Kornberg T.B., Prusiner S.B. (1995). Expression and targeting of Syrian hamster prion protein induced by heat shock in transgenic Drosophila melanogaster. Mech. Dev..

[114] Hamanaka T., Nishizawa K., Sakasegawa Y., Kurahashi H., Oguma A., Teruya K., Doh-ura K. (2015). Anti-prion activity found in beetle grub hemolymph of Trypoxylus dichotomus septentrionalis. Biochem. Biophys. Rep..

[115] International Platform of Insects for Food and Feed (2020). Guide on Good Hygiene Practices for European Union (EU) Producers of Insects as Food and Feed.

[116] Jensen A.N., Hansen S.H., Baggesen D.L. (2020). Salmonella Typhimurium Level in Mealworms (Tenebrio molitor) After Exposure to Contaminated Substrate. Front. Microbiol..

[117] Caparros Megido R., Poelaert C., Ernens M., Liotta M., Blecker C., Danthine S., Tyteca E., Haubruge É., Alabi T., Bindelle J. (2018). Effect of household cooking techniques on the microbiological load and the nutritional quality of mealworms (*Tenebrio molitor* L. 1758). Food Res. Int..

[118] Huis A.V. (2013). Potential of Insects as Food and Feed in Assuring Food Security. Annu. Rev. Entomol..

[119] Vandeweyer D., Lenaerts S., Callens A., Van Campenhout L. (2017). Effect of blanching followed by refrigerated storage or industrial microwave drying on the microbial load of yellow mealworm larvae (*Tenebrio molitor*). Food Control.

[120] Klunder H.C., Wolkers-Rooijackers J., Korpela J.M., Nout M.J.R. (2012). Microbiological aspects of processing and storage of edible insects. Food Control.

[121] Kröncke N., Grebenteuch S., Keil C., Demtröder S., Kroh L., Thünemann A.F., Benning R., Haase H. (2019). Effect of Different Drying Methods on Nutrient Quality of the Yellow Mealworm (*Tenebrio molitor* L.). Insects.

[122] Palsson P.G., Gissurarson M. (2016). HACCP Bókin—Fjölbreyttar og Gagnlegar Upplýsingar um HACCP og Framleiðslu Sjávarfangs (The HACCP Book—Diverse and Useful Information about HACCP and Seafood Production).

[123] Engstrom A. Är Det Lagligt Att Servera och sälja Insekter i Sverige? Här är Svaren! (Is It Legal to Serve and Sell Insects in Sweden? Here Are the Answers!). https://www.bugburger.se/politik/ar-det-lagligt-att-servera-insekter/?fbclid=IwAR1uixSogJqHPSp5nw68A5-S3eycvoFrkRiNHXUkQH13QACTy8qhjDOwqQo.

[124] International Platform of Insects for Food and Feed (2021). An Overview of the European Market of Insects as Feed.

[125] European Commission (2001). No 999/2001.

[126] European Commission (2011). Regulation (EU) No 189/2011.

[127] European Commission (2017). Regulation (EU) No 2017/893.

[128] European Commission (2021). Regulation (EU) No 2021/1372.

[129] Mancini S., Moruzzo R., Riccioli F., Paci G. (2019). European consumers’ readiness to adopt insects as food. A review. Food Res. Int..

[130] Schösler H., Boer J.D., Boersema J.J. (2012). Can we cut out the meat of the dish? Constructing consumer-oriented pathways towards meat substitution. Appetite.

[131] Naranjo-Guevara N., Fanter M., Conconi A.M., Flotè Stammen S. (2021). Consumer acceptance among Dutch and German students of insects in feed and food. Food Sci. Nutr..

[132] Sidali K.L., Pizzo S., Garrido-Pérez E.I., Schamel G. (2019). Between food delicacies and food taboos: A structural equation model to assess Western students’ acceptance of Amazonian insect food. Food Res. Int..

[133] Orsi L., Voege L.L., Stranieri S. (2019). Eating edible insects as sustainable food? Exploring the determinants of consumer acceptance in Germany. Food Res. Int..

[134] Lorini C., Ricotta L., Vettori V., Del Riccio M., Biamonte M.A., Bonaccorsi G. (2021). Insights into the Predictors of Attitude toward Entomophagy: The Potential Role of Health Literacy: A Cross-Sectional Study Conducted in a Sample of Students of the University of Florence. Int. J. Environ. Res. Public Health.

[135] Tuccillo F., Marino M.G., Torri L. (2020). Italian consumers’ attitudes towards entomophagy: Influence of human factors and properties of insects and insect-based food. Food Res. Int..

[136] International Platform of Insects for Food and Feed The European Insect Sector Is very Pleased about the Third EFSA Opinion on Edible Insects. https://ipiff.org/the-european-insect-sector-is-very-pleased-about-the-third-efsa-opinion-on-edible-insects/.

[137] International Platform of Insects for Food and Feed The European Insect Sector Welcomes the Latest EFSA Novel Food Opinion on Edible Insects!. https://ipiff.org/2021/07/.

[138] International Platform of Insects for Food and Feed IPIFF Welcomes the Fourth EFSA Opinion on Edible Insects. https://ipiff.org/ipiff-welcomes-the-fourth-efsa-opinion-on-edible-insects/.

[139] Kooistra J. (2020). Financial Feasibility Analysis of Insect Farming in the Netherlands. Ph.D. Thesis.

[140] International Platform of Insects for Food and Feed. https://ipiff.org.

[141] International Platform of Insects for Food and Feed IPIFF Members. https://ipiff.org/ipiff-members/.

[142] Turck D., Bresson J.-L., Burlingame B., Dean T., Fairweather-Tait S., Heinonen M., Hirsch-Ernst K.I., Mangelsdorf I., EFSA Panel on Dietetic Products, Nutrition and Allergies (NDA) (2016). Guidance on the preparation and presentation of an application for authorisation of a novel food in the context of Regulation (EU) 2015/2283. EFSA J..

[143] Garino C., Zagon J., Braeuning A. (2019). Insects in food and feed—Allergenicity risk assessment and analytical detection. EFSA J..

[144] Besnardeau E. (2021). Personal Communication.

[145] Hunt A. (2021). Personal Communication.

[146] Öhult M. (2021). Personal Communication.

[147] Kooistra J. (2021). Personal Communication.

[148] Marcenac X. (2021). Personal Communication.

[149] Leseultre X. (2021). Personal Communication.

[150] European Commision (2021). Comission Implementing Regulation (EU) 2021/882 of 1 June 2021. Off. J. Eur. Union.

[151] Food Standards Australia New Zealand (Te Mana Kounga Kai—Ahitereiria me Aotearoa) Supporting Document 4—Overeview of International Regulatory Approaches—Proposal P1024 Revision of the Regulation of Nutirive Substances & Novel Foods. https://www.foodstandards.gov.au/code/proposals/Documents/P1024%20Nuts%20and%20novels%20SD4%20International%20regs.pdf.

[152] IPIFF EU Legislation. https://ipiff.org/insects-eu-legislation/.

[153] European Commision Sustainable Developmental Goals. https://ec.europa.eu/international-partnerships/sustainable-development-goals_en.

[154] Behre E., Heukels B., Mayayo A.M., Verschuur X. (2017). Insects as Livestock Feed.

[155] European Commission Strategy. https://ec.europa.eu/info/strategy_en.

[156] European Commission (2019). Communication from the Commission to the European Parliament, the European Council, the Council, the European Economic and Social Committee and the Committee of the RegionS—The European Green Deal.

[157] BugBurger The Eatings Insects Startups: Here Is the List of Entopreneurs around. https://www.bugburger.se/foretag/the-eating-insects-startups-here-is-the-list-of-entopreneurs-around-the-world/.

[158] Van Huis A., Tomberlin J.K. (2017). Insects as Food and Feed: From Production to Consumption.

[159] Pikl D. (2021). Personal Communication.

[160] Rognstad T. (2021). Personal Communication.

[161] Munk-Bogballe D. (2021). Personal Communication.

[162] Horizon Insect LTD About Us. https://www.linkedin.com/company/horizon-insects-ltd.

[163] Tebrito About Us. https://www.linkedin.com/company/tebrito/.

[164] Entoinnov About Us. https://www.linkedin.com/company/entoinnov/.

[165] Enorm About Us. https://www.linkedin.com/company/enorm/.

[166] Hermetia Baruth GmbH About Us. https://be.linkedin.com/company/hermetia-baruth-gmbh?trk=similar-pages_result-card_full-click.

[167] EntoMass About Us. https://www.linkedin.com/company/entomass.

[168] NextAlim Welcome to NextAlim. https://www.nextalim.com.

[169] NextProtein NextProtein Feeding the Future. http://nextprotein.co.

[170] Illucens Gmbh Illucens. https://illucens.com/en/.

[171] HiProMine Company. https://hipromine.com/company/?lang=en.

[172] Micronutris About Us. https://www.linkedin.com/company/micronutris/.

[173] Entocycle About Us. https://www.linkedin.com/company/entocycle.

[174] Tebrio About Us. https://es.linkedin.com/company/tebri.

[175] Protix Food in Balance with Nature. https://protix.eu.

[176] Ortiz J.A.C., Ruiz A.T., Morales-Ramos J.A., Thomas M., Rojas M.G., Tomberlin J.K., Yi L., Han R., Giroud L., Jullien R.L., Dossey A.T., Morales-Ramos J.A., Rojas M.G. (2016). Chapter 6—Insect Mass Production Technologies. Insects as Sustainable Food Ingredients: Production, Processing and Food Applications.

[177] Pikl D. Beautiful Sunday Story about Cricky (Lijepa priča Nedjeljom) at Index.hr. https://cricky.eu/blog/beautiful-sunday-story-about-cricky-lijepa-prica-nedjeljom-at-index-hr/.

[178] Urbanmat Om Oss (About Us). https://www.urbanmat.no/om-oss/.

[179] Insectum About Us. https://insectum.farm/pages/about-insectum-aps.

[180] Crowe E. An Edible Insect Farm Has Launched in London. https://www.squaremeal.co.uk/restaurants/news/Edible-insect-farm-London-_9193.

[181] Jemthans S. Moraföretaget Tebrito—Årets Nytänkare Producerar Insektsprotein för Framtiden (The Parent Company Tebrito—This Year’s Innovator Produces Insect Protein for the Future). https://mora.se/aktuellt/lokalproducerad-mjolmask-kan-vara-framtidens-protein.

[182] EntoBreed EntoBreed—AgriTech Startup Voor Duurzame Meelwormenkweek (AgriTech Startup for Sustainable Mealworm Cultivation). https://entobreed.com.

[183] Marienlyst Ento Marienlyst Ento Organic Waste—Pure Food. https://www.marienlystento.dk.

[184] Micronutris Micronutris Créateur d’Alimentation Durable (Micronutris Creator of Sustainable Food). https://www.micronutris.com/en/home.

[185] Entoinnov Entoinnov We Are Changing the Paradigm of Insect Farming!. https://www.entoinnov.com/en/homepage-entoinnov/.

[186] Entocycle ENTOCYCLE. https://www.entocycle.com.

[187] Enorm Biofactory History & Vision. https://www.enormbiofactory.com/en/historie-og-vision.

[188] Tebrio. https://tebrio.com/en/.

[189] Bugimine. https://bugimine.com.

[190] Hermetia Futtermittel GbR (2013). Endbericht zum Forschungsvorhaben (Final Report on the Research Project).

[191] Entomass About Us. https://www.entomass.dk/about-us.

[192] PAPEK s.r.o https://www.insect-papek.eu/en/.

[193] Moruzzo R., Mancini S., Boncinelli F., Riccioli F. (2021). Exploring the Acceptance of Entomophagy: A Survey of Italian Consumers. Insects.

[194] Piha S., Pohjanheimo T., Lähteenmäki-Uutela A., Křečková Z., Otterbring T. (2018). The effects of consumer knowledge on the willingness to buy insect food: An exploratory cross-regional study in Northern and Central Europe. Food Qual. Prefer..

[195] Meyer-Rochow V.B., Kejonen A. (2020). Could Western Attitudes towards Edible Insects Possibly be Influenced by Idioms Containing Unfavourable References to Insects, Spiders and other Invertebrates?. Foods.

[196] Roma R., Ottomano Palmisano G., De Boni A. (2020). Insects as Novel Food: A Consumer Attitude Analysis through the Dominance-Based Rough Set Approach. Foods.

[197] Víur Hafa Hætt Starfsemi. http://viur.gyl.fi.

[198] Palsdottir G.H. Framleiðsla Skordýra Reyndist ekki Arðbær (Insect Production Was Not Profitable). https://www.bbl.is/frettir/framleidsla-skordyra-reyndist-ekki-ardbaer.

[199] Sverrisdottir S.M. Innleiða Skordýraát í Vestræna Menningu: Búi og Stefán Segja Frá Sinni Gulleggshugmynd (Implementing Insect Eating in Western Culture: Bui and Stefan Talk about Their Golden Egg Idea). https://studentabladid.com/efni/innleida-skordyraat-i-vestraena-menningu.

[200] Armannsson B. Framleiðendur Matvæla úr Skordýrum Þurfa Leyfi Frá ESB (Insect Food Producers Need License from the EU). https://www.visir.is/g/2016160209242.

[201] Benediktsdottir E.B. Rækta Mjölorma Heima Hjá Sér (Breed Mealworms in Their Home). https://www.ruv.is/frett/raekta-mjolorma-heima-hja-ser.

[202] Munu Skordýr Fæða Heiminn? (Will Insects Feed the World?). https://matis.is/frettir/munu-skordyr-faeda-heiminn/.

[203] Sigurdardottir K. Brauð úr Lirfum Krybba í Þróun Hjá MATÍS (Bread from Cricket’s Larvae in Development at Matis). https://www.ruv.is/frett/braud-ur-lirfum-krybba-i-throun-hja-matis.

[204] Hardarson S.M. (2021). Tilraunaræktun á Skordýrum til Manneldis eða Fóðurframleiðslu (Experimental Breeding of Insects for Human Consumption or Feed Production).

[205] Salomone R. (2017). Environmental impact of food waste bioconversion by insects: Application of Life Cycle Assessment to process using Hermetia illucens. J. Clean. Prod..

[206] Olafsson G. (2021). Personal Communication.

[207] Hauksdottir G. Cricket Snack to Go Jungle Bar on the Table in the USA. https://www.icenews.is/2016/01/28/cricket-snack-to-go-jungle-bar-on-the-table-in-the-usa/.

[208] The Reykavik Grapevine Clearer Legislation Needed for Insect Based Food. https://grapevine.is/news/2016/01/20/clearer-legislation-needed-for-insect-based-food/.

[209] Sturludottir E., Thorvaldsson G., Helgadottir G., Gudnason I., Sveinbjornsson J., Sigurgeirsson O.I., Sveinsson T. (2021). Fæðuöryggu á Íslandi. Skýrsla unnin fyrir atvinnuvega- og nýsköpunarráðuneytið (Food security in Iceland. Report prepared for the Ministry of Industry and Innovation). Rit LbhÍ.

[210] Saber D.A., Silka L. (2020). Food Waste as a Classic Problem that Calls for Interdisciplinary Solutions: A Case Study Illustration. J. Soc. Issues.

[211] Umhverfisstofnun Matarsóun á Íslandi (Food Waste in Iceland). http://matarsoun.is/default.aspx?pageid=929ad605-0b03-11e6-a224-00505695691b.

